# Supplementary material for: A non-archaeopterygid avialan theropod from the Late Jurassic of southern Germany
Source: eLife. 2019 May 14;8:e43789. doi: 10.7554/eLife.43789 (PMC6516837; doi:10.7554/eLife.43789)
Supplement: Supplementary file 1. [file elife-43789-supp1.docx]

**Supplementary file 1: List of characters used in phylogenetic analysis and phylogenetic data matrix**

**A non-archaeopterygid avialan theropod from the Late Jurassic of southern Germany**

Oliver W. M. Rauhut^1,2,3^* ^†^, Helmut Tischlinger^4^, and Christian Foth^5†^

^1^Staatliche naturwissenschaftliche Sammlungen Bayerns (SNSB), Bayerische Staatssammlung für Paläontologie und Geologie, Richard-Wagner-Str. 10, D-80333 München, Germany.

^2^Department for Earth and Environmental Sciences, Palaeontology & Geobiology, Ludwig-Maximilians-University, Richard-Wagner-Str. 10, D-80333 München, Germany.

^3^ GeoBioCenter, Ludwig-Maximilians-University, Richard-Wagner-Str. 10, D-80333 München, Germany.

^4^Tannenweg 16, D-85134 Stammham, Germany.

^5^Department of Geosciences, Université de Fribourg, Chemin du Musée 4, CH-1700 Fribourg, Switzerland

**Character list used for the phylogenetic analysis**

1. Vaned feathers on forelimb symmetric (0) or asymmetric (1).
2. Orbit round in lateral or dorsolateral view (0) or dorsoventrally elongate (1).
3. Anterior process of postorbital projects into orbit (0) or does not project into orbit (1).
4. Postorbital in lateral view with subhorizontal anterior (frontal) process (0) or frontal process diagonal (anterior tip of process higher than base of process) (1).
5. Postorbital bar parallels quadrate, lower temporal fenestra rectangular in shape (0) or jugal and postorbital approach or contact quadratojugal to constrict lower temporal fenestra (1).
6. Crista interfenestralis confluent with lateral surface of prootic and opisthotic (0) or distinctly depressed within middle ear opening (1).
7. Subotic recess (pneumatic fossa ventral to fenestra ovalis) absent (0) or present (1).
8. Basisphenoid recess present between basisphenoid and basioccipital (0) or entirely within basisphenoid (1) or absent (2).
9. Posterior opening of basisphenoid recess single (0) or divided into two small, circular foramina by a thin bar of bone (1).
10. Base of cultriform process not highly pneumatized (0) or base of cultriform process (parasphenoid rostrum) expanded and pneumatic (parasphenoid bulla) (1).
11. Basipterygoid processes ventral or anteroventrally projecting (0) or lateroventrally projecting (1) or laterally (2) (State 2 added after Turner et al. 2012).
12. Basipterygoid processes well developed, extending as a distinct process from the base of the basisphenoid (0) or processes abbreviated or absent (1).
13. Basipterygoid processes solid (0) or processes hollow (1).
14. Basipterygoid recesses on dorsolateral surfaces of basipterygoid processes absent (0) or present (1).
15. Depression for pneumatic recess on prootic absent (0) or present as dorsally open fossa on prootic/opisthotic (1) or present as deep, posterolaterally directed concavity (2). ORDERED.
16. Accessory tympanic recess dorsal to crista interfenestralis absent (0) or small pocket present (1) or extensive with indirect pneumatization (2). ORDERED.
17. Caudal (posterior) tympanic recess absent (0) or present as opening on anterior surface of paroccipital process (1) or extends into opisthotic posterodorsal to fenestra ovalis, confluent with this fenestra (2). ORDERED.
18. Exits of C.N. X-XII flush with surface of exoccipital (0) or cranial nerve exits located together in a bowl-like basisphenoid depression (1).
19. Maxillary process of premaxilla contacts nasal to form posterior border of nares (0) or maxillary process reduced so that maxilla participates broadly in external naris (1) or maxillary process of premaxilla extends posteriorly to separate maxilla from nasal posterior to nares (2).
20. Internarial bar rounded (0) or flat (1).
21. Crenulate margin on buccal edge of premaxilla absent (0) or present (1).
22. Caudal margin of naris farther rostral than (0) or nearly reaching or overlapping the rostral border of the antorbital fossa (1).
23. Premaxillary symphysis acute, V-shaped (0) or rounded, U-shaped (1).
24. Secondary palate short (0) or long, with extensive palatal shelves on maxilla (1).
25. Palatal shelf of maxilla flat (0) or with midline ventral tooth-like projection (1).
26. Pronounced, round accessory antorbital fenestra absent (0) or present (1).
27. Accessory antorbital fossa situated at rostral border of antorbital fossa (0) or situated posterior to rostral border of fossa (1).
28. Tertiary antorbital fenestra (fenestra promaxillaris) absent (0) or present (1).
29. Antorbital fossa without distinct rim ventrally and anteriorly (0) or with distinct rim composed of a thin wall of bone (1).
30. Narial region apneumatic or poorly pneumatized (0) or with extensive pneumatic fossae, especially along posterodorsal rim of fossa (1).
31. Jugal and postorbital contribute equally to postorbital bar (0) or ascending process of jugal reduced and descending process of postorbital ventrally elongate (1).
32. Jugal quadratojugal process tall beneath lower temporal fenestra, twice or more as tall dorsoventrally as it is wide transversely (0) or rod-like (1) or concealed by quadratojugal (2). ORDERED.
33. Jugal pneumatic recess in posteroventral corner of antorbital fossa present (0) or absent (1).
34. Medial jugal foramen present on medial surface ventral to postorbital bar (0) or absent (1).
35. Quadratojugal without horizontal process posterior to ascending process (reversed L shape) (0) or with process (i.e., inverted T or Y shape) (1).
36. Supraorbital crests on lacrimal in adult individuals absent (0) or dorsal crest above orbit (1) or lateral expansion anterior and dorsal to orbit (2).
37. Enlarged foramen or foramina opening laterally at the angle of the lacrimal, absent (0) or present (1).
38. Lacrimal posterodorsal process absent (inverted L shaped) (0) or posterior process present, but shorter than anterodorsal process (1) or posterior process as long as anterodorsal process (lacrimal T shaped in lateral view) (2) (modified).
39. Prefrontal large, dorsal exposure similar to that of lacrimal (0) or greatly reduced in exposure (1) or without exposure (2).
40. Frontals narrow anteriorly as a wedge between nasals (0) or end abruptly anteriorly, suture with nasal transversely orientated (1) or suture with nasals W-shaped (2).
41. Anterior emargination of supratemporal fossa on frontal straight or slightly curved (0) or strongly sinusoidal and reaching onto postorbital process (1).
42. Frontal postorbital process (dorsal view): smooth transition from orbital margin (0) or sharply demarcated from orbital margin (1).
43. Frontal edge smooth in region of lacrimal suture (0) or edge notched (1).
44. Dorsal surface of parietals flat, lateral ridge borders supratemporal fenestra (0) or parietals dorsally convex with very low sagittal crest along midline (1) or dorsally convex with well-developed sagittal crest (2). ORDERED.
45. Parietals separate (0) or fused (1).
46. Descending process of squamosal parallels quadrate shaft (0) or nearly perpendicular to quadrate shaft (1).
47. Descending process of squamosal contacts quadratojugal (0) or does not contact quadratojugal (1).
48. Posterolateral shelf on squamosal overhanging quadrate head absent (0) or present (1).
49. Quadrate vertical (0) or strongly inclined anteroventrally so that distal end lies far forward of proximal end (1).
50. Lateral border of quadrate shaft straight (0) or with lateral tab that touches squamosal and quadratojugal above an enlarged quadrate foramen (1).
51. Foramen magnum subcircular, slightly wider than tall (0) or oval, taller than wide (1).
52. Occipital condyle without constricted neck (0) or subspherical with constricted neck (1).
53. Paroccipital process elongate and slender, with dorsal and ventral edges nearly parallel (0) or process short, deep with convex distal end (1).
54. Paroccipital process straight, projects laterally or posterolaterally (0) or distal end curves ventrally, pendant (1).
55. Paroccipital process with straight dorsal edge (0) or with dorsal edge twisted rostrolaterally at distal end (1).
56. Ectopterygoid with constricted opening into fossa (0) or with open ventral fossa in the main body of the element (1).
57. Dorsal recess on ectopterygoid absent (0) or present (1).
58. Flange of pterygoid well developed (0) or reduced in size or absent (1).
59. Palatine and ectopterygoid separated by pterygoid (0) or contact (1).
60. Palatine tetraradiate, with jugal process (0) or palatine triradiate, jugal process absent (1).
61. Suborbital fenestra similar in length to orbit (0) or about half or less than half orbital length (1) or absent (2). ORDERED.
62. Symphyseal region of dentary broad and straight, paralleling lateral margin (0) or medially recurved slightly (1) or strongly recurved medially (2).
63. Dentary symphyseal region in line with main part of buccal edge (0) or symphyseal end downturned (1).
64. Mandible without coronoid prominence (0) or with coronoid prominence (1).
65. Dentary posteriorly unforked, or with a weakly developed dorsal ramus (0) or strongly forked with the dorsal and ventral rami approximately equal in posterior extent (1) (modified after Turner et al. 2012).
66. Labial face of dentary flat (0) or with lateral ridge and inset tooth row (1).
67. Nutrient foramina on external surface of dentary superficial (0) or lie within deep groove (1).
68. Surangular with a spinous rostral process (subdividing the external mandibular fenestra): absent (0) or present (1) (reworded).
69. Internal mandibular fenestra small and slit-like (0) or large and rounded (1).
70. Foramen in lateral surface of surangular rostral to mandibular articulation, absent (0) or present (1).
71. Splenial not widely exposed on lateral surface of mandible (0) or exposed as a broad triangle between dentary and angular on lateral surface of mandible (1).
72. Coronoid ossification large (0) or only a thin splint (1) or absent (2). ORDERED.
73. Articular without elongate, slender medial, posteromedial, or mediodorsal process from retroarticular process (0) or with process (1).
74. Retroarticular process short, stout (0) or elongate and slender (1).
75. Mandibular articulation surface as long as distal end of quadrate (0) or twice or more as long as quadrate surface, allowing anteroposterior movement of mandible (1).
76. Premaxilla toothed (0) or edentulous (1).
77. Second premaxillary tooth approximately equivalent in size to other premaxillary teeth (0) or second tooth markedly larger than third and fourth premaxillary teeth (1) or first premaxillary tooth considerably larger than the posterior ones (2).
78. Maxilla toothed (0) or edentulous (1).
79. Maxillary and dentary teeth serrated (0) or some without serrations anteriorly (except at base in S. mongoliensis) (1) or all without serrations (2).
80. Maxillary teeth large, less than 22 in (0) or large number of small teeth (≥ 22) (1) or small number of teeth (≤ 8) (2) (character modified).
81. Serration denticles large (0) or small (1).
82. Serrations simple, denticles convex (0) or distal and often mesial edges of teeth with large, hooked denticles that point toward the tip of the crown (1).
83. Teeth constricted between root and crown (0) or root and crown confluent (1).
84. Dentary teeth evenly spaced (0) or anterior dentary teeth smaller, more numerous, and more closely appressed than those in middle of tooth row (1).
85. Dentaries lack distinct interdental plates (0) or with interdental plates medially between teeth (1).
86. In cross section, premaxillary tooth crowns sub-oval to sub-circular (0) or asymmetrical (D-shaped in cross section) with flat lingual surface (1) or first premaxillary tooth with flat lingual surface, other premaxillary teeth without flat lingual surfaces (2).
87. Number of cervical vertebrae: 10 (0) or 12 or more (1).
88. Axial epipophyses absent or poorly developed, not extending past posterior rim of postzygopophyses (0) or large and posteriorly directed, extend beyond postzygapophyses (1).
89. Axial neural spine flared transversely (0) or compressed mediolaterally (1).
90. Epipophyses of cervical vertebrae placed distally on postzygapophyses, above postzygopophyseal facets (0) or placed proximally, proximal to postzygapophyseal facets (1).
91. Anterior cervical centra level with or shorter than posterior extent of neural arch (0) or centra extending beyond posterior limit of neural arch (1).
92. Carotid process on posterior cervical vertebrae absent (0) or present (1).
93. Anterior cervical centra subcircular or square in anterior view (0) or distinctly wider than high, kidney shaped (1).
94. Cervical neural spines anteroposteriorly long and dorsoventrally tall (0) or anteroposteriorly short, dorsoventrally low and centred on neural arch, giving arch an X shape in dorsal view (1) or anteroposteriorly short and dorsoventrally tall (2) or anteroposteriorly long and dorsoventrally short (3).
95. Cervical centra with one pair of pneumatic openings (0) or with two pairs of pneumatic openings (1).
96. Cervical and anterior trunk vertebrae amphiplatyan (0) or opisthocoelous (1) or at least partially heterocoelous (2) (State 2 was added from Turner et al. 2012).
97. Anterior trunk vertebrae without prominent hypapophyses (0) or with large hypapophyses (1).
98. Parapophyses of posterior trunk vertebrae flush with neural arch (0) or distinctly projected on pedicels (1).
99. Hyposphene -hypantrum articulations in trunk vertebrae absent (0) or present (1).
100. Zygapophyses of trunk vertebrae abutting one another above neural canal, opposite hyposphenes meet to form lamina (0) or zygapohyses placed lateral to neural canal and separated by groove for interspinuous ligaments, hyposphens separated (1).
101. Middle and posterior dorsal vertebrae not pneumatic (0) or pneumatic (1).
102. Transverse processes of anterior dorsal vertebrae long and thin (0) or short, wide, and only slightly inclined (1).
103. Neural spines of dorsal vertebrae not expanded distally (0) or expanded to form spine table (1).
104. Scars for interspinous ligaments terminate at apex of neural spine in dorsal vertebrae (0) or terminate below apex of neural spine (1).
105. Number of sacral vertebrae: 5 (0) or 6 (1) or 7 to 10 (2) or 11 or more (3) (State 2 and 3 newly added). ORDERED
106. Sacral vertebrae with unfused zygapophyses (0) or with fused zygapophyses forming a sinuous ridge in dorsal view (1).
107. Ventral surface of posterior sacral centra gently rounded, convex (0) or ventrally flattened, sometimes with shallow sulcus (1) or centrum strongly constricted transversely, ventral surface keeled (2).
108. Pleurocoels absent on sacral vertebrae (0) or present on anterior sacrals only (1) or present on all sacrals (2). ORDERED.
109. Last sacral centrum with flat posterior articulation surface (0) or convex articulation surface (1).
110. Caudal vertebrae with distinct transition point (0) or without transition point (1).
111. Transition point in caudal series begins distal to the 10th caudal (0) or between 7th and 10th caudal vertebra (1) or proximal to the 7th caudal vertebra (2).
112. Anterior caudal centra tall, oval in cross section (0) or with box-like centra in caudals I-V (1) or anterior caudal centra laterally compressed with ventral keel (2).
113. Neural spines of caudal vertebrae simple, undivided (0) or separated into anterior and posterior alae throughout much of caudal sequence (1).
114. Neural spines on distal caudals form a low ridge (0) or spine absent (1) or midline sulcus in center of neural arch (2).
115. Prezygapophyses of distal caudal vertebrae between 1/3 and whole centrum length (0) or with extremely long extensions of the prezygapophyses (up to 10 vertebral segments long in some taxa) (1) or strongly reduced as in Archaeopteryx lithographica (2).
116. More than 30 free caudal vertebrae (0) or 17-30 free caudal vertebrae (1) or < free 17 caudal vertebrae (2) (modified). ORDERED.
117. Proximal end of chevrons of proximal caudals short anteroposteriorly, shaft proximodistally elongate (0) or proximal end elongate anteroposteriorly, flattened and plate-like (1).
118. Distal caudal chevrons are simple (0) or anteriorly bifurcate (1) or bifurcate at both ends (2).
119. Shaft of cervical ribs slender and longer than vertebra to which they articulate (0) or broad and shorter than vertebra (1).
120. Ossified uncinate processes absent (0) or present (1) or fused to ribs (2) (State 2 was added from Turner et al. 2012). ORDERED.
121. Ossified ventral rib segments absent (0) or present (1).
122. Lateral gastral segment shorter than medial one in each arch (0) or distal segment longer than proximal segment (1).
123. Ossified sternal plates separate in adults (0) or fused (1) or absent (2) (State 3 newly added).
124. Sternum without distinct lateral xiphoid process posterior to costal margin (0) or with lateral xiphoid process (1).
125. Anterior edge of sternum grooved for reception of coracoids (0) or sternum without grooves (1).
126. Articular facet of coracoid on sternum (conditions may be determined by the articular facet on coracoid in taxa without ossified sternum): anterolateral or more lateral than anterior (0) or almost anterior (1).
127. Hypocleidium on furcula absent (0) or present as tubercle (1) or present as elongate process (2) (State 2 was added from Turner et al. 2012). ORDERED.
128. Acromion margin of scapula continuous with blade (0) or anterior edge laterally everted or hooked (1) (State 1 modified).
129. Anterior surface of coracoid ventral to glenoid fossa unexpanded (0) or anterior edge of coracoid expanded, forms triangular subglenoid fossa bounded laterally by coracoid tuber (1).
130. Scapula and coracoid separate (0) or fused into scapulacoracoid (1).
131. Coracoid in lateral view subcircular, with shallow ventral blade (0) or subquadrangular with extensive ventral blade (1) or shallow ventral blade with elongate posteroventral process (2) or subtriangular (proximal end constricted, distal end wide) and strut-like (3) (State 3 modified).
132. Scapula and coracoid form a continuous arc in posterior and anterior views (0) or coracoid inflected medially, scapulocoracoid L-shaped in lateral view (1).
133. Glenoid fossa without (0) or with extension of glenoid floor onto external surface of scapula (the surface opposite the costal surface) (1).
134. Scapula longer than humerus (0) or humerus longer than scapula (1).
135. Deltopectoral crest large and distinct, proximal end of humerus quadrangular in anterior view (0) or deltopectoral crest less pronounced, forming an arc rather than being quadrangular (1) or deltopectoral crest very weakly developed, proximal end of humerus with rounded edges (2) or deltopectoral crest extremely long (3) or proximal end of humerus extremely broad, triangular in anterior view (4).
136. Anterior surface of deltopectoral crest smooth (0) or with distinct groove or ridge near lateral edge along distal end of crest (1).
137. Olecranon process weakly developed (0) or distinct and large but not hypertrophied (1) or hypertrophied (2). ORDERED.
138. Distal articular surface of ulna flat (0) or convex, semilunate surface (1).
139. Proximal surface of ulna with a single continuous articular facet (0) or divided into two distinct fossae separated by a median ridge (1).
140. Lateral proximal carpal (ulnare?) quadrangular (0) or triangular in proximal view (1).
141. Two distal carpals in contact with metacarpals, one covering the base of metacarpal I (and perhaps contacting metacarpal II) the other covering the base of metacarpal II (distal carpals 1 and 2 unfused) (0) or a single distal carpal capping metacarpals I and II (distal carpals 1 and 2 fused) (1).
142. Distal carpals 1+2 well developed, covering all of proximal ends of metacarpals I and II (0) or small, cover about half of base of metacarpals I and II (1) or cover bases of all metacarpals (2) or covers metacarpals II and III (3).
143. Metacarpal I half or less than half the length of metacarpal II, and longer proximodistally than wide transversely (0) or subequal in length to metacarpal II (1) or very short and wider transversely than long proximodistally (2).
144. Third manual digit present, phalanges present (0) or reduced to no more than metacarpal splint (1).
145. Flexor tubercles of manual unguals proximal (0) or displaced distally from articular end (1) or proximodistally elongated with proximal end close to articular facet (2).
146. Unguals on all digits generally similar in size (0) or digit I bearing large ungual and unguals of other digits distinctly smaller (1).
147. Proximodorsal lip on first manual ungual (a transverse ridge immediately dorsal to the articulating surface) absent (0) or present (1).
148. Ventral edge of anterior ala of ilium straight or gently curved (0) or ventral edge hooked anteriorly (1) or very strongly hooked (2).
149. Preacetabular part of ilium roughly as long as postacetabular part of ilium (0) or preacetabular portion of ilium markedly longer (more than 2/3 of total ilium length) than postacetabular part (1).
150. Anterior end of ilium gently rounded or straight (0) or anterior end strongly curved (1) or pointed at anterodorsal corner (2) or with notch at anterodorsal corner (3).
151. Supraacetabular crest on ilium as a separate process from antitrochanter, forms hood over femoral head present (0) reduced, not forming hood (1) or absent (2). ORDERED.
152. Postacetabular ala of ilium in lateral view squared (0) or acuminate (1).
153. Postacetabular blades of ilia in dorsal view parallel (0) or diverge posteriorly (1).
154. Tuber along dorsal edge of ilium, dorsal or slightly posterior to acetabulum absent (0) or present (1).
155. Brevis fossa shelf-like (0) or deeply concave with lateral overhang (1).
156. Antitrochanter posterior to acetabulum absent or poorly developed (0) or prominent (1).
157. Ridge bordering cuppedicus fossa extends far posteriorly and is confluent or almost confluent with acetabular rim (0) or ridge terminates rostral to acetabulum or curves ventrally onto anterior end of pubic peduncle (1).
158. Cuppedicus fossa deep, ventrally concave (0) or fossa shallow or flat, with no lateral overhang (1) or absent (2). ORDERED.
159. Proximodorsal process of ischium small, tablike or pointed process along caudal edge of ischium (0) or process large proximodorsally hooked and separated from iliac peduncle of the ischium by a notch (1) (modified after Turner et al. 2012).
160. Shaft of ischium straight in lateral view (0) or ventrodistal end curved anteriorly (1) or curved dorsally (posterodorsally concave) (2).
161. Obturator process of ischium absent (0) or proximal in position (1) or located near middle of ischiadic shaft or distally (2) (State 2 modified). ORDERED.
162. Obturator process does not contact pubis (0) or contacts pubis (1).
163. Length of pubic boot ≤ 30% length of pubis (0) or ≥ 40% (1).
164. Semicircular scar on posterior part of the proximal end of the ischium, absent (0) or present (1).
165. Ischium, ischium length as measured by ischial length/pubic length ratio: more than 70% (0) or between 50%-70% (1) or below 50% (2) (modified). ORDERED.
166. Distal ends of ischia form symphysis (0) or approach one another but do not form symphysis (1) or widely separated (2). ORDERED.
167. Ischial boot (expanded distal end) present (0) or absent (1).
168. Tubercle on anterior edge of ischium absent (0) or present (1).
169. Pubis propubic (0) or pubis vertical (1) or pubis moderately posteriorly oriented (2) or pubis fully posteriorly oriented (opisthopubic) (3). ORDERED.
170. Pubic boot projects anteriorly and posteriorly (0) or with little or no anterior process (1) or no anteroposterior projections (2).
171. Shelf on pubic shaft proximal to symphysis (= pubic apron) extends medially from middle of cylindrical pubic shaft (0) or shelf extends medially from anterior edge of anteroposteriorly flattened shaft (1) or absent (2) (State 2 added from Turner et al. 2012).
172. Pubic shaft straight (0) or distal end curves anteriorly, anterior surface of shaft concave in lateral view (1) or anterior surface of shaft convex in lateral view (2).
173. Pubic apron about half of pubic shaft length (0) or less than 1/3 of shaft length (1).
174. Femoral head without fovea capitalis (for attachment of capital ligament) (0) or circular fovea present in center of medial surface of head (1).
175. Lesser and greater trochanters unfused (0) or trochanters separated by small groove (1) or completely fused (or absent) to form a trochantic crest (2).
176. Lesser trochanter of femur alariform (0) or cylindrical in cross section (1).
177. Lateral ridge absent or represented only by rugose area (0) or posterior trochanter distinctly raised from shaft, mound -like or shelf-like (1).
178. Fourth trochanter on femur present (0) or absent (1).
179. Accessory trochanteric crest distal to lesser trochanter absent (0) or present (1).
180. Anterior surface of femur proximal to medial distal condyle without longitudinal crest (0) or crest present extending proximally from medial condyle on anterior surface of shaft (1).
181. Popliteal fossa on distal end of femur open distally (0) or closed off distally by contact betweeen distal condyles (1).
182. Fibula reaches proximal tarsals (0) or short, tapering distally, and not in contact with proximal tarsals (1).
183. Medial surface of proximal end of fibula concave along long axis (0) or flat (1).
184. Deep oval fossa on medial surface of fibula near proximal end absent (0) or present (1).
185. Distal end of tibia and astragalus without distinct condyles (0) or with distinct condyles separated by prominent tendinal groove on anterior surface (1).
186. Ascending process of the astragalus tall and broad, covering most of anterior surface of distal end of tibia (0) or process short and slender, covering only lateral half of anterior surface of tibia (1) or ascending process tall with medial notch that restricts it to lateral side of anterior face of distal tibia (2).
187. Ascending process of astragalus confluent with condylar portion (0) or separated by transverse groove or fossa across base (1).
188. Astragalus and calcaneum separate from tibia (0) or fused to each other but unfused to tibia (1) or completely fused to each other and the tibia (2). ORDERED.
189. Distal tarsals separate, not fused to metatarsals (0) or form metatarsal cap with intercondylar prominence that fuses to metatarsal early in postnatal ontogeny (1).
190. Metatarsals not co-ossified (0) or co-ossification of metatarsals begins proximally (1) or metatarsals fuse to each other proximally and distally (2) or extreme distal fusion, distal vascular foramen closed (3). ORDERED.
191. Distal end of metatarsal II smooth, not ginglymoid (0) or with developed ginglymus (1).
192. Distal end of metatarsal III smooth, not ginglymoid (0) or with developed ginglymus (1).
193. Metatarsal III, proximal end: not pinched (0) or pinched, but visible in anterior view (1) or pinched, not exposed along proximal section of metapodium (2) or proximal part of metatarsal III lost (3).
194. Ungual of pedal digit II similar in size to that of III (0) or pedal ungual II about 50% larger than pedal ungual III (1).
195. Metatarsal I articulates at middle of metatarsal II (0) or metatarsal I attaches to distal quarter of metatarsal II (1) or metatarsal I articulates with metatarsal II near its proximal end (2) or metatarsal I absent (3).
196. Metatarsal I attenuates proximally (0) or proximal end of metatarsal I similar to that of metatarsals II-IV (1).
197. Shaft of MT IV round or thicker dorsoventrally than wide in cross section (0) or shaft of MT IV mediolaterally widened and flat in cross section (1).
198. Foot symmetrical (0) or asymmetrical with slender MTII and very robust MT IV (1) or asymmetrical with MT IV more slender than MT II (2) (state 2 added).
199. Neural spines on posterior dorsal vertebrae in lateral view rectangular or square (0) or anteroposteriorly expanded distally, fanshaped (1).
200. Shaft diameter of phalanx I-1 less (0) or greater (1) than shaft diameter of radius.
201. Angular exposed almost to end of mandible in lateral view, reaches or almost reaches articular (0) or excluded from posterior end angular suture turns ventrally and meets ventral border of mandible rostral to glenoid (1).
202. Laterally inclined flange along dorsal edge of surangular for articulation with lateral process of lateral quadrate condyle absent (0) or present (1).
203. Distal articular ends of metacarpals I + II ginglymoid (0) or rounded, smooth (1) or II ginglymoid and metacarpal I shelf (2).
204. Radius and ulna well separated (0) or with distinct adherence or syndesmosis distally (1).
205. Quadrate head covered by squamosal in lateral view (0) or quadrate cotyle of squamosal open laterally exposing quadrate head (1).
206. Brevis fossa poorly developed adjacent to ischial peduncle and without lateral overhang, medial edge of brevis fossa visible in lateral view (0) or fossa well developed along full length of postacetabular blade, lateral overhang extends along full length of fossa, medial edge completely covered in lateral view (1).
207. Vertical ridge on lesser trochanter present (0) or absent (1).
208. Supratemporal fenestra bounded laterally and posteriorly by the squamosal (0) or supratemporal fenestra extended as a fossa on to the dorsal surface of the squamosal (1).
209. Dentary fully toothed (0) or only with teeth rostrally (1) or edentulous (2).
210. Posterior edge of coracoid not or shallowly indented below glenoid (0) or posterior edge of coracoid deeply notched just ventral to glenoid, glenoid lip everted (1).
211. Retroarticular process points caudally (0) or curves gently dorsocaudally (1).
212. Flange on supraglenoid buttress on scapula (see Nicholls and Russell, 1985) absent (0) or present (1).
213. Depression (possibly pneumatic) on ventral surface of postorbital process of laterosphenoid absent (0) or present (1).
214. Basal tubera set far apart, level with or beyond lateral edge of occipital condyle and/or foramen magnum (may connected by a web of bone or separated by a large notch) (0) or tubera small, directly below condyle and foramen magnum, and separated by a narrow notch (1) or absent (2).
215. Basioccipital without pneumatization on occipital surface (0) or with subcondylar recess (1).
216. Distal humerus with small or no medial epicondyle (0) or with large medial epicondyle, medial condyle centered on distal end (1).
217. Distal humeral condyles on distal end (0) or on anterior surface (1).
218. Ilium and ischium articulation flat or slightly concavo-convex (0) or ilium with process projecting into socket in ischium (1).
219. Roots of dentary and maxillary teeth mediolaterally compressed (0) or circular in cross-section (1).
220. Preacetabular portion of ilium parasagital (0) moderately laterally flaring (1) strongly laterally flaring (2). ORDERED.
221. Maxillary and dentary teeth labiolingually flattened and recurved, with crowns in middle of tooth row more than twice as high as the basal mesiolateral width (0) or lanceolate and subsymmetrical (1) or conical (2) or labiolingually flattened and recurved, with crowns in middle of tooth row less than twice as high as the basal mesiolateral width (3).
222. Dentary teeth do not (0) or do increase in size anteriorly, becoming more conical in shape (1).
223. Length of skull more than 90% femoral length (0) or less than 80% (1).
224. Height of skull (minus mandible) at middle of naris more than half the height of skull at middle of orbit (0) or less than half (1).
225. Dorsal margin of naris below level of dorsal margin of orbit (0) or above (1).
226. Snout does not (0) or does taper to an anterior point (1).
227. Area of antorbital fenestra greater than that of orbit (0) or less than that of orbit (1).
228. Shape of the premaxillary body: wider than high, or approximately as wide as high (0) or significantly higher than wide (1).
229. Antorbital fossa anteriorly bounded by maxilla (0) or by premaxilla (1).
230. Maxillary antorbital fossa: small, from 10% to less than 40% of the rostrocaudal length of the antorbital cavity (0) or large, greater than 40% of the rostrocaudal length of the antorbital cavity (1).
231. Maxillary fenestra large and round (0) or a large, craniocaudally elongate oblong (1) or a small, craniocaudally elongate slit, not dorsally displaced (2) or a small, dorsally displaced opening (3).
232. Nasal fusion: absent, nasals separate (0) or present, nasals fused together (1).
233. Nasal surface: smooth (0) or rugose (1).
234. Suborbital process of jugal short and dorsoventrally stout (0) or elongate and dorsoventrally narrow (1).
235. Nasals at least as long as frontals (0) or shorter than frontals (1).
236. Jugo-maxillary bar at ventral end of antorbital fenestra dorsoventrally deep (0) or dorsoventrally narrow (1).
237. Anteroventral corner of premaxilla does not (0) or does form an acute, ventrally orientated point in lateral view (1).
238. Length of preorbital region of cranium > height at anterior edge of preorbital bar (exclusive of midline sagittal ridge, if any) (0) or ≤ height at anterior edge of preorbital bar (1).
239. Frontals without supraorbital rim (0) or with supraorbital rim (1).
240. Parietals shorter than frontals (0) or longer (1).
241. Length of ventral border of infratemporal fenestra comparable to that of orbit (0) or much shorter (1).
242. Foramen magnum smaller than or subequal to size of occipital condyle (0) or larger than occipital condyle (1).
243. Dorsal margin of the dentary: straight or concave (0) or convex (1) or sigmoid (2) in lateral view (character modified).
244. Meckelian groove of dentary deep (0) or shallow (1).
245. Dentary without posteroventral process extending to posterior end of external mandibular fenestra (0) or with such a process (1).
246. Horizontal shelf on the lateral surface of the surangular, rostral and ventral to the mandibular condyle: absent or faint ridge (0) or prominent and extending laterally (1).
247. Premaxillary teeth subequal in size to (0) or much smaller than (1) the maxillary teeth.
248. Approximately the same number of denticles per 5 mm on mesial keels of teeth as on distal keels (0) or markedly more denticles per 5 mm on mesial keels (1).
249. Dentary tooth implantation: in sockets (0) or in paradental groove (1).
250. Dentary dentition continues cranially to tip of dentary (0) or terminates before reaching dentary tip (1).
251. Length of mid-cervical centra approximately the same as dorsal centra (0) or markedly longer than dorsal centra (1).
252. Cervical prezygapophyses unflexed (0) or flexed (1).
253. Dorsal centra ≥ 1.2x taller than long (0) or height ≤ length (1).
254. Posterior dorsal neural spines ≥ 1.5x taller than long (0) or height < 1.5x length (1).
255. Postzygapophyses of middle and posterior dorsal vertebrae do not extend posterior to centrum (0) or do (1).
256. Anteriormost haemal arches ≥ 1.5x longer than associated centra (0) or < 1.5x as long as centra (1).
257. Angle between furcular arms greater than or equal 90° (0) or less than 90° (1) (modified from Turner et al. 2012).
258. Scapula, acromion process projected anteriorly to surpass the articular surface for coracoid (facies articularis coracoidea) (0) or projected less anteriorly than the articular surface for coracoid (1).
259. Acromion process: significantly and abruptly expanded dorsally (0) or rise gradually from the scapular blade (1).
260. Wide distal expansion of scapula absent (0) or present (1).
261. Acrocoracoid process absent or poorly developed (0) or prominent developed and straight (1) or prominent and medially hooked (2).
262. Humeral length is half femoral length or less (0) or shorter than femur but more than half femoral length (1) or longer than femur (2). ORDERED.
263. Length of humeral shaft between deltopectoral crest and distal condyles < 4.5x shaft diameter (0) or > 4.5x shaft diameter (1).
264. Ulna not bowed away from radius (0) or bowed away from radius (1).
265. Length of radius < 1/3 femoral length (0) or between 1/3 and 2/3 femoral length (1) or between 2/3 and 1x femoral length (2) or > femoral length (3). ORDERED.
266. Radial diameter > 0.5x ulnar diameter (0) or ≤ 0.5x (1).
267. Distal carpals 1+2 flattish (0) or semilunate in shape (1).
268. Length of manual digit II (including metacarpal) less than 1.25 x femoral length (0) or 1.25x femoral length (1).
269. Distal end of metacarpal I medially (0) or laterally rotated (1).
270. Medial side of metacarpal II: expanded proximally (0) or not expanded (1).
271. Metacarpal III < 0.8x length of metacarpal II (0) or 0.8-1x length of metacarpal II (1) or metacarpal III longer than metacarpal II (2) (modified). ORDERED.
272. Manual phalanx I-1 longer than metacarpal II (0) or shorter (1).
273. Length of metacarpal II < length of metacarpal I + phalanx I-1 (0) or ≥ (1).
274. Metacarpals II and III are not (0) or are appressed for their entire lengths (1).
275. Proximal end of metacarpal III is not (0) or is mainly palmar to that of metacarpal II (1).
276. Length of manual phalanx II-2 < 1.2x length of phalanx II-1 (0) or 1.2-1.9x length of phalanx II-1 (1) or ≥ 2x length of phalanx II-1 (2). ORDERED.
277. Medial ligament pits of manual phalanges deep (0) or shallow (1).
278. Posterior flange on manual phalanx II-1 absent (0) or present (1).
279. Combined lengths of manual phalanges II-1 and II- 2 > length of metacarpal II + carpus (0) or ≤ length of metacarpal II + carpus (1).
280. Length of manual phalanx II-1 < 2x length of III-1 (0) or ≥ 2x length of III-1 (1).
281. Length of manual phalanx III-1, sub-equal to III-2 (0) or considerably longer (1) or considerably shorter (2).
282. Manual phalanx I-1 straight (0) or bowed (palmar surface concave) (1).
283. With proximal articular surface of ungual orientated vertically, dorsal surface of manual ungual I does not (0) or does arch higher than level of dorsal extremity of proximal articular surface (1).
284. With proximal articular surface of ungual orientated vertically, dorsal surface of manual ungual II does not (0) or does arch higher than level of dorsal extremity of proximal articular surface (1).
285. Manual ungual I strongly curved (0) or weakly curved (1) or straight (2). ORDERED.
286. Manual unguals II and III strongly curved (0) or weakly curved (1) or straight (2). ORDERED.
287. Proximodorsal lip on manual unguals II and III absent (0) or present (1).
288. Manual digit III with four phalanges (0) or less than four phalanges (1).
289. Manual phalanx III-3 markedly shorter than combined lengths of phalanges III-1 and III-2 (0) or subequal in length to their combined lengths (1) or markedly longer (2). ORDERED.
290. Arching of preacetabular iliac blade above height of postacetabular blade absent or small (0) or extreme (1).
291. Shaft of ischium slenderer than the pubic shaft (0) or subequal in thickness to the pubis (1) or thicker than the pubic shaft (2). ORDERED.
292. Obturator process does not (0) or does form a strongly acute angle in lateral view (1).
293. Ventral notch between the distal portion of the obturator process and the shaft of the ischium: present (0) or absent (1).
294. Strong kink of pubis at midshaft absent (0) or present, displacing distal half of pubis caudally (1).
295. In adult, femur longer than tibia (0) or shorter (1).
296. Tip of lesser trochanter below level of femoral head (0) or level with femoral head (1).
297. Metatarsus less than half length of femur (0) or more than half femoral length (1) or longer than femur (2). ORDERED.
298. Metatarsal cross-sectional proportions: subequal or wider mediolaterally than craniocaudally at midshaft (0) or deeper craniocaudally than mediolaterally at midshaft (1).
299. Shafts of metatarsals not appressed (0) or appressed (1).
300. Length of metatarsal V ≥ 0.5x length of metatarsal IV (0) or < 0 .5x (1) or absent (2). ORDERED.
301. Marked decrease in transverse width of metatarsus distally, absent (0) or present (1).
302. Plantar surface of hallux faces posteriorly (0) or hallux reorientated so that plantar surface faces medially or anteriorly (1).
303. Hallucal ungual reduced in size relative to other pedal unguals (0) or not reduced (1).
304. Hallucal ungual weakly curved (0) or strongly curved (1).
305. Length of pedal phalanx II-2 length ≤ 0.6x of phalanx II-1 (0) or between 0.6 and 1x (1) or ≥ 1x (2). ORDERED.
306. Total length of pedal phalanx II-2 (not counting posteroventral lip, if any) > 2x length of distal condylar eminence (0) or ≤ 2x (1).
307. Pedal phalanx II-2 without posteroventral lip or heel (0) or with transversely narrow posteroventral heel (1) or with transversely wide posteroventral lip (2). ORDERED.
308. Pedal phalanx II-1 without dorsal extension of distal condyles (0) or with extension (1).
309. Pedal unguals III and IV straight or weakly curved (0) or strongly curved (1).
310. With fingers extended, tip of ungual III extends no further distally than flexor tubercle of ungual II (0) or extends further (1).
311. Manual ungual III smaller than ungual II (0) or approximately the same size (1).
312. Diameter of non-ungual phalanges of manual digit III > 0.5x diameter of non-ungual phalanges of digit II (0) or < 0.5x (1).
313. Manual phalanx II-1 shorter than I-1 (0) or longer (1).
314. Ischial shaft rodlike (0) or flat, platelike (1).
315. Lateral face of ischial shaft flat (or round in rodlike ischia) (0) or laterally concave (1) or with longitudinal ridge dividing lateral surface into anterior and posterior parts (2).
316. Contact between pubic apron contributions of both pubes meet extensively (0) or contact interrupted by a slit (1) or no contact (2).
317. Dorsal margin of postacetabular iliac blade straight or convex (0) or concave (1).
318. Large, longitudinal flange along caudal or lateral face of metatarsal IV absent (0) or present (1).
319. Distally placed dorsal process along caudal edge of ischial shaft absent (0) or present (1).
320. Length of metatarsus < 3.5x transverse midshaft diameter (0) or 3.5-8x midshaft diameter (1) or > 8x midshaft diameter (2). ORDERED
321. Lengths of mid-caudal centra subequal to or less than those of proximal caudal centra (0) or more than twice as long as proximal caudal centra (1).
322. Pubic peduncle of ilium craniocaudally longer (0) or shorter (1) than ischial peduncle of ilium.
323. Phalanges of pedal digit III not blocky (proximal phalanx length ≥ 2x diameter) (0) or blocky (proximal phalanx length < 2x diameter) (1).
324. Width of distal humeral expansion < 1/3 humeral length (0) or ≥ 1/3 humeral length (1).
325. Lateral epicondyle of humerus not expanded laterally (0) or expanded laterally (1).
326. Distal end of metatarsal I reduced in size relative to distal ends of other metatarsals (0) or comparable in size to distal ends of other metatarsals (1).
327. Pedal phalanx II-1 longer (0) or shorter (1) than pedal phalanx IV-1.
328. Dentary ramus elongate (0) or shortened, not much longer than tall (1).
329. Metacarpal II ≥ 1/3 humeral length (0) or < 1/3 humeral length (1).
330. With fingers extended, tip of ungual I does not extend past flexor tubercle of ungual II (0) or extends past flexor tubercle of ungual II but does not extend past tip of ungual II (1) or extends past tip of ungual II (2). ORDERED
331. Premaxillary teeth serrated (0) or unserrated (1).
332. Sublacrimal part of jugal tapering (0) or bluntly squared anteriorly (1) or expanded (2) or bifurcated (3).
333. Flexor tubercles of manual unguals ≥ 1/3x height of articular facet (0) or < 1/3 (1).
334. Distal chevrons straight or L-shaped in lateral view (0) or upside-down T-shaped (1).
335. Metacarpal III distally not ginglymoid (0) or ginglymoid (1).
336. Breadth of acromion process perpendicular to long axis of scapular blade: deep (0) or shallow (1).
337. Proximal end of metatarsal IV curls around plantar side of proximal end of metatarsal III (0) or does not (1).
338. Midsagittal ridge formed by dorsal displacement of midline of frontals, nasals and premaxillae, absent (0) or present (1).
339. Ectopterygoid lateral to pterygoid (0) or rostral to pterygoid (1).
340. Palatine-pterygoid-ectopterygoid bar does not (0) or does (1) arch below ventral cheek margin.
341. Co-ossification of angular and surangular absent (0) or present (1).
342. Cervical ribs unfused to cervical vertebrae (0) or fused to cervical vertebrae (1).
343. Anterior caudal vertebrae without pneumatopores (0) or with pneumatopores (1).
344. External mandibular fenestra not rostrally displaced (sits beneath orbit) (0) or rostrally displaced (sits largely anterior to orbit) (1).
345. Ilium, pubic peduncle: substantially larger than (0) or subequal to (1) ischial peduncle.
346. Ischium, shape: distally narrower (0) or distally wider (1) (excluding obturator process).
347. Humerus, thickness relative to femur: much thinner (0) or subequal (1).
348. Promaxillary fenestra, exposure in lateral view: minimal (0) or significant (1).
349. Antorbital fossa, shape: anteroposterior diameter greater (0) or less (1) than dorsoventral diameter.
350. Antorbital fenestra, size relative to external naris: larger (0) or smaller (1).
351. Jugal, postorbital process, location: considerably anterior to the posterior end of the jugal (0) or nearly at the posterior end so that the quadratojugal process is minimal (1).
352. External mandibular fenestra, size: small or absent (0) or large (1).
353. Ilium, preacetabular process: deep (0) or shallow (1).
354. Lacrimal, posterodorsal process, orientation: subvertical (0) or posteriorly inclined (1).
355. Anterior caudal vertebrae, transverse processes, distal tapering: absent (0) or present (1).
356. Anteroproximal contact between metatarsals II and IV absent (0) or present (1).
357. Pennaceous feathers: absent (0) or present (1).
358. Cross-section of pedal ungual III and IV triangular (0) or vertically oval (1).
359. Accessory antorbital (maxillary) fenestra recessed within a shallow, caudally or caudodorsally open fossa, which is itself located within the maxillary antorbital fossa: absent (0) or present (1).
360. In lateral view, dorsal border of the antorbital fossa formed by the lacrimal and maxilla (0) or by the lacrimal and nasal (1) or by maxilla, premaxilla, and lacrimal (2).
361. Obturator notch present (0) or notch or foramen absent (1).
362. Jugal and quadratojugal separate (0) or quadratojugal and jugal fused and not distinguishable from one another (1).
363. Tibia, cnemial crest(s) lateral crest only (0) or lateral and anterior crests developed (1).
364. Postacetabular end of ilium terminating in rounded or square end in dorsal view (0) or with lobate brevis shelf projecting from end of ilium and beyond end of postacetabular lamina (1).
365. Lateral face of pubic shaft smooth (0) or with prominent lateral tubercle about halfway down the shaft (1).
366. Triangular obturator process with short rostral projection and wide base along ischial shaft (0) or short base, long process extending rostrally (1).
367. Tuber along extensor surface metatarsal II (associated with the insertion of the tendon of the m. tibialis cranialis in Aves) absent (0) or present, on approximately the center of the proximodorsal surface of metatarsal II (1) or present, developed on lateral surface of metatarsal II, at contact with metatarsal III or on lateral edge of metatarsal III (2). ORDERED.
368. Nasal process of maxilla, dorsal ramus (ascending ramus of maxilla): prominent, exposed medially and laterally (0) or absent or reduced to slight medial, and no lateral exposure (1).
369. In lateral view, participation of the ventral ramus of the nasal process of the maxilla in the anterior margin of the internal antorbital fenestra present extensively (0) or small dorsal projection of the maxilla participates in the anterior margin (1) or no dorsal projection of maxilla participates in the anterior margin (2).
370. In lateral view, dorsal border of the internal antorbital fenestra formed by lacrimal and maxilla (0) or lacrimal and nasal (1).
371. In lateral view, lateral lamina of the ventral ramus of nasal process of maxilla present, large broad exposure (0) or present, reduced to small triangular exposure (1).
372. Supratemporal fossa with limited extension onto dorsal surfaces of frontal and postorbital (0) or covers most of frontal process of the postorbital and extends anteriorly onto dorsal surface of frontal (1).
373. Jugal does not particulate in margin of antorbital fenestra (0) or participates in antorbital fenestra (1).
374. Maxillary tooth height highly variable with gaps evident for replacement (0) or almost isodont with no replacement gaps (1).
375. Splenial forms notched anterior margin of internal mandibular fenestra (0) or present (1).
376. Nasal dorsally flat for most of length (0) or dorsally convex (1).
377. Squamosal-quadratojugal flange constricting infratemporal fenestra absent (0) or present (1).
378. Supraoccipital, pronounced, strongly demarcated median ridge absent (0) or present (1).
379. Surangular, posterior surangular foramen small (0) or large fenestra (1).
380. Vertical ridge on iliac blade above acetabulum absent or poorly developed (0) or well developed (1).
381. Axial neural spine sheetlike (0) or anteroposteriorly reduced and rodlike (1).
382. Humerus in lateral view sigmoidal (0) or straight (1).
383. Ulna less than half the length of humerus (0) or more than half the length of humerus, but shorter than the humerus (1) or ulna approximately the same length or significantly longer than humerus (2). ORDERED.
384. Premaxillae unfused in adults (0) or fused anteriorly in adults, posterior nasal [frontal] processes not fused to each other (1) or frontal processes completely fused as well as anterior premaxillae (2). ORDERED.
385. Dentaries joined proximally by ligaments (0) or joined by bone (1).
386. Mandibular symphysis, two strong grooves forming an anteriorly opening V in ventral view absent (0) or present (1).
387. Facial margin primarily formed by the maxilla, with the maxillary process of the premaxilla restricted to the anterior tip (0) or maxillary process of the premaxilla extending ½ facial margin (1) or maxillary process of the premaxilla extending more than ½ of facial margin (2). ORDERED.
388. Nasal [frontal] process of premaxilla short (0) or long, closely approaching frontal (1).
389. Ectopterygoid present (0) or absent (1).
390. Articulation between vomer and pterygoid present, well developed (0) or reduced, narrow process of pterygoid passes (1) or dorsally over palatine to contact vomer (1) or absent, pterygoid and vomer do not contact (2).
391. Palatine and pterygoid long, anteroposteriorly overlapping, contact (0) or short, primarily dorsoventral, contact (1).
392. Palatine contacts maxillae only (0) or premaxillae and maxillae (1).
393. Vomer contacts premaxilla present (0) or absent (1).
394. Projecting basisphenoid articulation with pterygoid present (0) or absent (1).
395. Basisphenoid-pterygoid articulations located basal on basisphenoid (0) or located markedly anterior on basisphenoid (parasphenoid rostrum) such that the articulations are subadjacent on the narrow rostrum (1).
396. Basisphenoid-pterygoid articulation, orientation of contact anteroventral (0) or mediolateral (1) or entirely dorsoventral (2).
397. Pterygoid, articular surface for basisphenoid concave ‘‘socket’’ or short groove enclosed by dorsal and ventral flanges (0) or flat to convex (1) or flat to convex facet, stalked, variably projected (2). ORDERED.
398. Pterygoid, kinked present, surface for basisphenoid articulation at high angle to axis of palatal process of pterygoid (0) or absent, articulation in line with axis of pterygoid (1).
399. Osseous interorbital septum (mesethmoid) absent (0) or present (1).
400. Osseous interorbital septum (mesethmoid) restricted to posterior or another just surpassing premaxillae/frontal contact in rostral extent does not surpass posterior edge of external nares in rostral extent (0) or extending rostral to posterior extent of frontal processes of premaxillae and rostral to posterior edge of external nares (1).
401. Eustachian tubes paired and lateral (0) or paired, close to cranial midline (1) or paired and adjacent on midline or single anterior opening (2).
402. Eustachian tubes ossified absent (0) or present (1).
403. Orbital process of quadrate, pterygoid articulation pterygoid broadly overlapping medial surface of orbital process (i.e., ‘‘pterygoid ramus’’) (0) or restricted to anteromedial edge of process (1).
404. Quadrate, orbital process pterygoid articulates with anteriormost tip (0) or pterygoid articulation does not reach tip (1) or pterygoid articulation with no extent up orbital process, restricted to quadrate corpus (2). ORDERED.
405. Quadrate/pterygoid contact as a facet, variably with slight anteromedial projection cradling base (0) or condylar, with a well-projected tubercle on the quadrate (1).
406. Quadrate, well-developed tubercle on anterior surface of dorsal process absent (0) or present (1).
407. Quadrate, quadratojugal articulation overlapping (0) or peg and socket articulation (1).
408. Quadrate, dorsal process, articulation with squamosal only (0) or with squamosal and prootic (1).
409. Quadrate, dorsal process, development of intercotylar incisure between prootic and squamosal cotylae absent, articular surfaces not differentiated (0) or two distinct articular facets, incisure not developed (1) or incisure present, ‘‘double headed’’ (2).
410. Quadrate, mandibular articulation bicondylar articulation with mandible (0) or tricondylar articulation, additional posterior condyle or broad surface (1).
411. Quadrate, pneumaticity absent (0) or present (1).
412. Quadrate, cluster of pneumatic foramina on posterior surface of the tip of dorsal process absent (0) or present (1).
413. Quadrate, pneumatization, large, single pneumatic foramen absent (0) or posteromedial surface of corpus (1).
414. Articular pneumaticity absent (0) or present (1).
415. Splenial, anterior extent splenial stops well posterior to mandibular symphysis (0) or extending to mandibular symphysis, though noncontacting (1) or extending to proximal tip of mandible, contacting on midline (2).
416. Mandibular symphysis, anteroposteriorly extensive, flat to convex, dorsalfacing surface developed absent, concave (0) or flat surface developed (1).
417. Mandibular symphysis, symphyseal foramina absent (0) or present (1).
418. Mandibular symphysis, symphyseal foramen/foramina single (0) or paired (1).
419. Mandibular symphysis, symphyseal foramen/foramina opening on posterior edge of symphysis (0) or opening on dorsal surface of symphysis (1).
420. Jugal/postorbital contact present (0) or absent (1).
421. Frontal/parietal suture open (0) or fused (1).
422. Thoracic vertebrae, count 12 or more (0) or 11 (1) or 10 or fewer (2). ORDERED.
423. Thoracic vertebrae at least part of series with subround, central articular surfaces (e.g., amphicoelous/opisthocoelous) that lack the dorsoventral compression seen in heterocoelous vertebrae (0) or series completely heterocoelous (1).
424. Thoracic vertebrae, parapophyses rostral to transverse processes (0) or directly ventral to transverse processes (close to midpoint of vertebrae) (1).
425. Thoracic vertebrae, centra, length, and midpoint width approximately equal in length and midpoint width (0) or length markedly greater than midpoint width (1).
426. Thoracic vertebrae with ossified connective tissue bridging transverse processes absent (0) or present (1).
427. Notarium absent (0) or present (1).
428. Sacral vertebrae, series of short vertebrae, with dorsally directed parapophyses just anterior to the acetabulum absent (0) or present, 3 such vertebrae (1) or present, 4 such vertebrae (2). ORDERED.
429. Anterior Free caudals prior to transition point; length of transverse processes subequal to width of centrum (0) or significantly shorter than centrum width (1).
430. Distal caudals unfused (0) or fused (1).
431. Fused distal caudals, morphology fused element length equal or greater than 4 free caudal vertebrae (0) or length less than 4 caudal vertebrae (1) or less than 2 caudal vertebrae in length (2). ORDERED.
432. Gastralia present (0) or absent (1).
433. Carina or midline ridge absent (0) or slightly raised (1) or distinctly projected (2).
434. Carina or midline ridge restricted to posterior half of sternum (0) or approaches anterior limit of sternum (1) or restricted to the anterior half of the sternum (2).
435. Sternum, dorsal surface, pneumatic foramen (or foramina) absent (0) or present (1).
436. Sternum, pneumatic foramina in the depressions (loculi costalis) between rib articulations (processi articularis sternocostalis) absent (0) or present (1).
437. Sternum, coracoidal sulci spacing on anterior edge widely separated mediolaterally (0) or adjacent (1) or crossed on midline (2).
438. Sternum, number of processes for articulation with the sternal ribs 3 (0) or 4 (1) or 5 (2) or 6 (3) or 7 or more (4). ORDERED.
439. Sternum: raised, paired intermuscular ridges (linea intermuscularis) parallel to sternal midline absent (0) or present (1).
440. Sternum, posterior margin, distinct posteriorly projected medial and/or lateral processes absent (directly laterally projected zyphoid processes developed but not considered homologes as these are copresent with the posterior processes in the new clade) (0) or with distinct posterior processes (1) or midpoint of posterior sternal margin connected to medial posterior processes to enclose paired fenestra (2). ORDERED.
441. Furcula, laterally excavated absent (0) or present (1).
442. Furcula, dorsal (omal) tip flat or blunt tip (0) or with a pronounced posteriorly pointed tip (1).
443. Furcula, ventral margin of apophysis curved, angled (0) or with a truncate or squared base (1).
444. Scapula and coracoid articulation pit-shaped scapular cotyla developed on the coracoid, and coracoidal tubercle developed on the scapula (‘‘ball and socket’’ articulation) (0) or scapular articular surface of coracoid convex (1) or flat (2).
445. Coracoid, procoracoid process absent (0) or present (1).
446. Coracoid, lateral margin straight to slightly concave (0) or convex (1).
447. Coracoid, dorsal surface (= posterior surface of basal maniraptoran theropods) strongly concave (0) or flat to convex (1).
448. Coracoid, pneumatized absent (0) or present (1).
449. Coracoid, pneumatic foramen proximal (0) or distal (1).
450. Coracoid, lateral process absent (0) or present (1).
451. Coracoid, ventral surface, lateral intermuscular line or ridge absent (0) or present (1).
452. Coracoid, glenoid facet dorsal to, or at approximately same level as, acrocoracoid process/‘‘biceps tubercle’’ (0) or ventral to acrocoracoid process (1).
453. Coracoid, n. supracoracoideus passes through coracoid present (0) or absent (1).
454. Coracoid, medial surface, area of the foramen n. supracoracoideus (when developed) strongly depressed (0) or flat to convex (1).
455. Scapula straight (0) or dorsoventrally curved (1).
456. Humerus, proximal end, head in anterior or posterior view straplike, articular surface flat, no proximal midline convexity (0) or head domed proximally (1).
457. Humerus, proximal end, proximal projection dorsal edge projected farthest (0) or midline projected farthest (1).
458. Humerus, ventral tubercle and capital incisure absent (0) or present (1).
459. Humerus, capital incisures an open groove (0) or closed by tubercle associated with a muscle insertion just distal to humeral head (1).
460. Humerus, anterior surface, well-developed fossa on midline making proximal articular surface appear V-shaped in proximal view absent (0) or present (1).
461. Humerus, ‘‘transverse groove’’ absent (0) or present, developed as a discreet, depressed scar on the proximal surface of the bicipital crest or as a slight transverse groove (1).
462. Humerus, deltopectoral crest less than shaft width (0) or same width (1) or dorsoventral width greater than shaft width (2). ORDERED.
463. Humerus, deltopectoral crest, proximoposterior surface flat to convex (0) or concave (1).
464. Humerus, deltopectoral crest not perforate (0) or with a large fenestra (1).
465. Humerus, bicipital crest, pit-shaped scar/fossa for muscular attachment on anterodistal, distal or posterodistal surface of crest absent (0) or present (1).
466. Humerus, bicipital crest, pit-shaped fossa for muscular attachment anterodistal on bicipital crest (0) or directly ventrodistal at tip of bicipital crest (1) or posterodistal, variably developed as a fossa (2).
467. Humerus, bicipital crest little or no anterior projection (0) or developed as an anterior projection relative to shaft surface in ventral view (1) or hypertrophied, rounded tumescence (2). ORDERED.
468. Humerus, proximal end, one or more pneumatic foramina absent (0) or present (1).
469. Humerus, long axis of dorsal condyle at low angle to humeral axis, proximodistally orientated (0) or at high angle to humeral axis, almost transversely orientated (1).
470. Humerus, distal condyles subround, bulbous (0) or weakly defined, ‘‘straplike’’ (1).
471. Humerus, distal margin approximately perpendicular to long axis of humeral shaft (0) or ventrodistal margin projected significantly distal to dorsodistal margin, distal margin angling strongly ventrally (sometimes described as a well-projected flexor process) (1).
472. Humerus, distal end, compressed anteroposteriorly and flared dorsoventrally absent (0) or present (1).
473. Humerus, brachial fossa absent (0) or present, developed as a flat scar or as a scarimpressed fossa (1).
474. Humerus, ventral condyle length of long axis of condyle less than the same measure of the dorsal condyle (0) or same or greater than same measure of the dorsal condyle (1).
475. Humerus, demarcation of muscle origins (e.g., m. extensor metacarpi radialis in Aves) on the dorsal edge of the distal humerus no indication of origin as a scar, a pit, or a tubercle (0) or indication as a pit-shaped scar or as a variably projected scar-bearing tubercle or facet (1).
476. Humerus, distal end, posterior surface, groove for passage of m. scapulotriceps absent (0) or present (1).
477. Humerus, m. humero tricipitalis groove absent (0) or present as a ventral depression contiguous with the olecranon fossa (1).
478. Ulna, cotylae dorsoventrally adjacent (0) or widely separated by a deep groove (1).
479. Ulna, dorsal cotyla convex absent (0) or present (1).
480. Ulna, distal end, dorsal condyle, dorsal trochlear surface, extent along posterior margin less than transverse measure of dorsal trochlear surface (0) or approximately equal in extent (1).
481. Ulna, bicipital scar absent (0) or developed as a slightly raised scar (1) or developed as a conspicuous tubercle (2). ORDERED.
482. Ulna, brachial scar absent (0) or present (1).
483. Radius, ventroposterior surface smooth (0) or with muscle impression along most of surface (1) or deep longitudinal groove (2).
484. Ulnare absent (0) or present (1).
485. Ulnare ‘‘heart shaped,’’ little differentiation into short dorsal and ventral rami (0) or V-shaped, well-developed dorsal and ventral rami (1).
486. Ulnare, ventral ramus (crus longus) shorter than dorsal ramus (crus brevis) (0) or same length as dorsal ramus (1) or longer than dorsal ramus (2).
487. Semilunate carpal and metacarpals no fusion (0) or incomplete proximal fusion (1) or complete proximal fusion (2) or complete proximal and distal fusion (3). ORDERED.
488. Metacarpal III, anteroposterior diameter as a percent of same dimension of metacarpal II approximately equal or greater than 50% (0) or less than 50% (1).
489. Metacarpal I, anteroproximally projected muscular process absent no distinct process visible (0) or small knob at anteroproximal tip of metacarpal (1) or tip of process just surpasses the distal articular facet for phalanx 1 in anterior extent (2) or tip of extensor process conspicuously surpasses articular facet by approximately half the width of facet, producing a pronounced knob (3) or tip of extensor process conspicuously surpasses articular facet by approximately the width of facet, producing a pronounced knob (4). ORDERED.
490. Metacarpal I, anterior surface roughly hourglass shaped proximally, at least moderately expanded anteroposteriorly, and constricted just before flare of articulation for phalanx 1 (0) or anterior surface broadly convex (1).
491. Pisiform process absent (0) or present (1).
492. Carpometacarpus, ventral surface, supratrochlear fossa deeply excavating proximal surface of pisiform process absent (0) or present (1).
493. Intermetacarpal space (between metacarpals II and III) reaches proximally as far as the distal end of metacarpal I (0) or terminates distal to end of metacarpal I (1).
494. Intermetacarpal process or tubercle absent (0) or present as scar (1) or present as tubercle or flange (2). ORDERED.
495. Manual digit II, phalanx 1 subcylindrical to subtriangular (0) or strongly dorsoventrally compressed, flat caudal surface (1).
496. Manual digit II, phalanx 2, internal index process on posterodistal edge absent (0) or present (1).
497. Ilium, ischium, pubis, proximal contact in adult unfused (0) or partial fusion (pubis not ankylosed) (1) or completely fused (2). ORDERED.
498. Ilium/ischium, distal coossification to completely enclose the ilioischiadic fenestra absent (0) or present (1).
499. Ischium, dorsal process does not contact ilium (0) or contacts ilium (1).
500. Laterally projected process on ischiadic peduncle (antitrochanter) directly posterior to acetabulum (0) or posterodorsal to acetabulum (1).
501. Ilium, preacetabular pectineal process absent (0) or present as a small flange (1) or present as a well-projected flange (2). ORDERED.
502. Preacetabular ilium approach on midline, open, or cartilaginous connection (0) or coossified, dorsal closure of ‘‘iliosynsacral canals’’ (1).
503. Preacetabular ilium extends anterior to first sacral vertebrae no free ribs overlapped (0) or one or more ribs overlapped (1).
504. Postacetabular ilium dorsoventrally orientated (0) or mediolaterally orientated (1).
505. Postacetabular ilium, ventral surface, renal fossa developed absent (0) or present (1).
506. Pubis suboval in cross section (0) or compressed mediolaterally (1).
507. Pubes, distal contact contacting, variably coossified into symphysis (0) or noncontacting (1).
508. Femur, patellar groove absent (0) or present (1).
509. Femur, ectocondylar tubercle and lateral condyle separated by deep notch (0) or form single trochlear surface (1).
510. Femur, posterior projection of the lateral border of the distal end, continuous with lateral condyle absent (0) or present (1).
511. Laterally projected fibular trochlea absent (0) or present, developed as small notch (1) or a shelflike projection (2). ORDERED.
512. Tibia/tarsal formed condyles medial condyle projecting further anteriorly than lateral (0) or equal in anterior projection (1).
513. Tibia/tarsal formed condyles, extensor canal absent (0) or an emarginated groove (1) or groove bridged by an ossified supratendoneal bridge (2). ORDERED.
514. Tibia/tarsal formed condyles, tuberositas retinaculi extensoris (Baumel and Witmer, 1993) indicated by short medial ridge or tubercle proximal to the condyles close to the midline and a more proximal second ridge on the medial edge absent (0) or present (1).
515. Tibia/tarsal formed condyles, mediolateral widths medial condyle wider (0) or approximately equal (1) or lateral condyle wider (2). ORDERED.
516. Tibia/tarsal formed condyles gradual sloping medial constriction of condyles (0) or no medial tapering of either condyle (1).
517. Tibia/tarsal formed condyles, intercondylar groove mediolaterally broad, approximately 1/3 width of anterior surface (0) or less than 1/3 width of total anterior surface (1).
518. Tibia, extension of articular surface for distal tarsals/tarsometatarsus no posterior extension of trochlear surface, or restricted to distalmost edge of posterior surface (0) or well-developed posterior extension, sulcus cartilaginis tibialis of Aves (Baumel and Witmer, 1993), distinct surface extending up the posterior surface of the tibiotarsus (1) or with well-developed, posteriorly projecting medial and lateral crests (2). ORDERED.
519. Tibia, distalmost mediolateral width wider than midpoint of shaft, giving distal profile a weakly developed triangular form approximately equal to shaft width (0), no distal expansion of whole shaft, although condyles may be variably splayed mediolaterally (1).
520. Metatarsal III proximally in plane with II and IV (0) or proximally displaced plantarly, relative to metatarsals II and IV (1).
521. Tarsometatarsus, intercotylar eminence absent (0) or well developed, globose (1).
522. Tarsometatarsus, projected surface or grooves on proximoposterior surface (associated with the passage of tendons of the pes flexors in Aves; hypotarsus) absent (0) or developed as posterior projection with flat posterior surface (1) or projection, with distinct crests and grooves (2) or at least one groove enclosed by bone posteriorly (3). ORDERED.
523. Tarsometatarsus, proximal vascular foramen (foramina) absent (0) or one, between metatarsals III and IV (1) or two (2). ORDERED.
524. Metatarsal I straight (0) or curved or distally deflected but not twisted, ventral surface convex ‘‘J shaped’’ (1) or deflected and twisted such that the ventromedial surface is concave proximal to trochlear surface for phalanx I (2). ORDERED.
525. Metatarsal II, distal plantar surface, fossa for metatarsal I (fossa metatarsi I) absent (0) or shallow notch (1) or conspicuous ovoid fossa (2). ORDERED.
526. Metatarsals, comparative trochlear width II approximately the same size as III and/or IV (0) or II wider than III and/or IV (1) or II narrower than III and/or IV (2) or IV narrowest (3).
527. Distal vascular foramen simple, with one exit (0) or forked, two exits (plantar and distal) between metatarsals III and IV (1).
528. Metatarsal III, trochlea in plantar view, proximal extent of lateral and medial edges of trochlea absent, trochlear edges approximately equal in proximal extent (0) or present, lateral edge extends further (1).
529. Metatarsal II, distal extent of metatarsal II relative to metatarsal IV approximately equal in distal extent (0) or metatarsal II shorter than metatarsal IV, but reaching distally further than base of metatarsal IV trochlea (1) or metatarsal II shorter than metatarsal IV, reaching distally only as far as base of metatarsal IV trochlea (2). ORDERED.
530. Middle to posterior caudal vertebrae 2x or less the length of dorsal vertebrae (0) or 3x–4x length of dorsal vertebrae (1).
531. Metacarpal III straight (0) or bowed (1).
532. Metatarsal I distal end of trochlea proximally placed relative to other metatarsals (0) or inline distally with others (1).
533. Metatarsal I present (0) or absent (1).
534. Development of the preotic pendent absent (0) or present but small (1) or present and robust (2). ORDERED.
535. Shape of the metotic strut short and robust (0) or long and narrow (1).
536. Prootic recess absent (0) present and shallow (1) or present and deep (2). ORDERED.
537. Anterior tympanic recess (ATR) absent (i.e., not deeply impressed into the lateral wall of basisphenoid) (0) or present and impressed into the lateral wall of the basisphenoid (1).
538. Location of ATR and the anterior tympanic crista below cranial nerve VII exit just proximal to the otic recess (0) or anteriorly with little or no development posterior to the basipterygoid processes (1).
539. ATR confluent with the subotic recess absent (0) or present, forming the lateral depression (1).
540. V-shaped opening between basal tubera remnants absent (0) or present (1).
541. Small tubera (not basal tubera) medial to basal tubera (or basal tubera remnants) and ventral to occipital condyle absent (0) or present (1).
542. Pedal phalanx II-2, distal articular surface relative to proximal articular surface approximately equal in size, distal surface slightly smaller than proximal (0) or distal surface less than half the size of proximal surface (1).
543. Ulna, size of proximal cotylae unequal, lateral (dorsal in birds) smaller (0) or equal (1).
544. Middle ear resides within the lateral depression absent (0) or present (1).
545. Quadratojugal size large (0) or greatly reduced (1).
546. Notch for postorbital contact on postorbital process of frontal absent, process smooth or facet small (0) or large notch present (1).
547. Position of frontoparietal suture relative to postorbital processes of frontal well posterior to the postorbital processes (0) or at the level of the postorbital processes (1) or anterior to postorbital processes (2).
548. Orientation of articular surfaces between cervical vertebrae surfaces vertical to subvertical (0) or strongly slanted anteroventrally (1).
549. Accessory depression in supratemporal fossa absent (0) or present (1).
550. Relative ventral extension of pubic versus ischiadic peduncles equal (0) or pubic peduncle extends farther ventrally (1).
551. Ala parasphenoidalis absent (0) or present, well developed and crest shaped, forming anterior edge of enlarged pneumatic recess with the ala continuous with the anterior tympanic crista (1).
552. Cross section of the furcula nearly circular (0) or anteroposteriorly compressed near the symphysis (1).
553. General shape of the furcula V-shaped (0) or U-shaped (1).
554. Epicledial processes unexpanded (0) or expanded (1).
555. Lateral expansion of the rami between the hypocledium and the epicledial process absent (0) or present (1).
556. Hypocledium rounded (0) or keeled (1).
557. Furcula asymmetrical (0) or nearly symmetrical (1).
558. Furcula rami thin (0) or thick (1).
559. Accessory longitudinal ridge on anterolateral side of the distal end of metatarsal IV absent (0) or present (1).
560. Extensive large pennaceous feathers on metatarsus and pes: absent (0), or present (1).
561. Longitudinal furrows on medial and lateral side of manual non-ungual phalanges: absent (0), present (1).
562. NEW: Attachment for m. pectoralis on deltopectoral crest of humerus: not specifically marked, distal edge of deltopectoral crest might be slightly expanded (0); marked as an elongate oval, anteromedially inclined facet on the mediodistal surface of the deltopectoral crest (1).
563. NEW: Proximal articular surface of ulna: anteroposteriorly concave and flat or slightly convex transversely (0); developed as an oval concavity with slightly raised rims (1).
564. NEW: Tuberculum bicipitale radii on the proximal radius: absent or indistinct (0); pronounced as a marked tubercle or crest (1).
565. NEW: Manual phalanx II-1: not significantly broadened when compared to other manual phalanges (0); strongly broadened, more than 1.5 times the width of phalanx II-2 and phalanges of digit III (1).

**Data matrix**

#NEXUS

BEGIN TAXA;

TITLE Taxa;

DIMENSIONS NTAX=136;

TAXLABELS

Allosaurus Sinraptor Albertosaurus Ajancingenia Anserimimus Archaeornithomimus Alxasaurus Alvarezsaurus Albinykus Avimimus Austroraptor Adasaurus Atrociraptor Achillobator Almas Archaeopteryx Alcmonavis Apsaravis Anas Beipiaosaurus Buitreraptor Byronosaurus Balaur Baptornis Bambiraptor Coelurus Compsognathus Caudipteryx Chirostenotes Citipati Conchoraptor Confuciusornis Cathayornis Concornis Chauna Crypturellus Crax Daspletosaurus Dilong Deinocheirus Deinonychus Dromaeosaurus Eotyrannus Erlikosaurus Erliansaurus Elmisaurus Eosinopteryx Falcarius Guanlong Gorgosaurus Gallimimus Garudimimus Graciliraptor Gobipteryx Gallus Huaxiagnathus Harpymimus Haplocheirus Hagryphus Heyuannia Hesperonychus Hongshanornis Hesperornis Incisivosaurus 'IGM100/44' 'IGM100/1126' Iaceornis Ichthyornis Jinfengopteryx Khaan Limenavis Liaoningornis Lithornis Mahakala Mei Mononykus Microvenator Microraptor Nanshiungosaurus Neimongosaurus Nothronychus Neuquenornis Ornitholestes Ornithomimus Oviraptor Ostromia Proceratosaurus Pelecanimimus Parvicursor Patagonykus Protarchaeopteryx Pyroraptor Pedopenna Pengornis Patagopteryx Rinchenia Rahonavis Sinosauropteryx Shenzhousaurus Sinornithomimus Struthiomimus Segnosaurus Shuvuuia Similicaudipteryx Saurornitholestes Sinornithoides Saurornithoides Sinusonasus Sinovenator Sinornithosaurus Songlingornis Tanycolagreus Tarbosaurus Tyrannosaurus Therizinosaurus Tianyuraptor Tsaagan Troodon Utahraptor Unenlagia Velociraptor Vorona Xixiasaurus Xiaotingia Yixianosaurus Yanornis Yixianornis Zanabazar Jixiangornis Epidexipteryx Jinguofortis Zhenyuanlong Jianianhualong Anchiornis Jeholornis Sapeornis;

END;

BEGIN CHARACTERS;

DIMENSIONS NCHAR=565;

FORMAT DATATYPE = STANDARD GAP = - MISSING = ? SYMBOLS = " 0 1 2 3 4 5";

MATRIX

Allosaurus ?1100?0000000?0001{0 1}00100010000001111100201?20000000100010000000000000100000000001010100100000001001000000000000010000000012???000000000010000?00010100000010000010100000000000000011000001000000000000000000100000000000000000000000000000000000000001000000000001000000000000100000001000000000001000000000000010000001000100000000000000020000000000000?0000000?00?00100000?000000001000001010000002000000000?000??00000000000???00000?000{0 1}0?0????????00020?00?0000100?0?000000?000000000000?0???10?01000??00000?000?000000000000000000000?0?0000002000?0000?0001000000{0 1}0?000?00000

Sinraptor ?1100??00?000?000100000001010000001110020102?000000000010000?0000000?10?0000000010101001000000010010000000000000??????????1??0?0?0??00??0??????000010000?0100?00100000000100000000010000010000000000000?100?00?00?00000??00000000000000000000000000001000000100??100????0??0?01??00?0???????00???020000000010?0010000??0?0010000?00??000??021?000000000000??00000?00?00100000?0000011010???01??0000000000000000?000??0000000?0000??000001000???00200??00???2??????????0????????????????????????????????1?????0?000??000000000000000000000000?0?00?0002000???00?00010000?????????0????

Albertosaurus ?1000?00?0000?002?0?00110??1?00?0001101?0?021?0?000?00000000?0?????0?1???0000000101011?????0????001?10??0????000??0????????????000000010000???01?00103100010000010?1011000000?0?00?0000??010000020000000100000000000?0??0????????0?1???11??0??????0????00000?????1?100??00??????0???????????????????1????????000??0?0????00?000????????0???2?????????????????0????????00100000?0000110?111111100000000???0000000000??00000101?000??00000?00??0?0?????????0?20010?0000101?0?000000?00000000000010??0?????000??00?00?0000000010000000000000000?0??00??02000??000?000100000110?000??????

Ajancingenia ?01?0???????????????1111110?010?1?1?122??0?1???00?0011?????122111001100?0111?1????????????0??1??1???1???1???01????2100???0{0 1}11121011?000000??1000010100201??11?022000011010?110110100000?00110000000000?1?000?11?2000?0?000?0??101011110?01?1110?012?10?????????01110010110100111000000100000011000101011101?00?????00100010100000000000101?0010110110011000?1101?0?0?1??1?00000??????????000?01?100000??1?????0?0?0??0000?000??10??00?0????0{0 1}0??02??????010?0??0???00?00?????100???0?0?????????????????00?????0?00?000?0?00?0?0?000?0?00??00?????0000????????01000?1?0?1101001???0??0

Anserimimus ???????????????????????????????????????????????????????????????????????????????????????????????????0?????????????????0??????????1???0?2????00?10100100000011000110?????000?00???????????????00002?3?00????11?10??1?1???????????????????????????????????????????????1???00?0?1?1001110001000022001???1?????1?0?????????1000010002???00???11??1?0????????????????????1????0??0000?????????????????????????????????????????????????????????????????????????????0???????0?????????????????????????????????010000?000???????????????????????0000??2??0?0?1???????????????????????????0???0

Archaeornithomimus ???????????????????????????????????????????????????????????????????????????????????????00??0?1?0001000001?100000000?0??????????010200?20100?01{0 1}0100??000011?00011001000000?0?0000?1000??0??0000010300000?111100?1111?0?000?????????????????????????????????1110??1??1?1000??111?0002000???001200??101010?11?0?????0????0000?000100?00???11??10001????00?000?????0?01????1?0?000???????????????????????????????????????????????????????00100?{0 1}0?????????????20?00?0000??1?0?000000?000000000000?0000???0?0000?00?00?????00000000000000000000??0?0?0????????????0????0????????????0????

Alxasaurus ?????????????????????????????????????????????????????????????210?10???????????0100001??????????0?01000000?1?00???0210?1??????????????00000?1000000020021?00?0100210?021?2?????01????00???????00000210000??00?1??00?0???1??1111????????????????????10???0011111?????0?100101?010?000?1?????1?001??1?01???0???????10000??0?1??000?010001?0????000???????0?000??????????1??1??00?0?????????????????0???????????????????????????????0?????0??0??{0 1}0??????????????0???????0?0??????0?0???0??????????????????0000???00?????0????????????????????????????00?0????0???0???????0??????????00??0

Alvarezsaurus ?????????????????????????????????????????????????????????????????????????????????????????1???100????????0?2?10?2?12?0??????????0?0?00????????0??1??000111?0??2????????????????1?00???0?01?10000000???0???????1???0?????????0??????????????????????????????111110??1???????????????????????0?1?????????????1?????100?0???????0??1000???0?????11??1????00???????????00?0?????0??0????????????0??????????????????????????????????????????0?1???00?????????????2????????0?0?????????????????????????????????????????0??0?0?00??????0000000????????????????????????????????????????0??????

Albinykus ???????????????????????????????????????????????????????????????????????????????????????????????????????????????????????????????????????????????????????1?????????????????????????????1101??11100300000?????????????????????????????????????????????????????????????????????????????????????????????????????1??????0?????????00?????????????????????????????????????????????0?????????????????????????????????????????????????????????????????????????????????????????????????????????????????????????????????????0??????0??????01000010?000000??0??00????????0????????????????1??????

Avimimus ?01?0??10011?00??0??1?1????????1????????00?11??00100110??????2?1??00?0??0111???????????011010110101?0?00??100????????????????????1?000100???10?????00?211?01??002000?1?010100001100?0????011110030???00??0?001?02?0000000??0?????0?1?????1????1011???0????001????????1110?1??????0????????????????101011101?0???10000????1010002?0000?0?????????10??01?0000?????1??1?0??1100000????0????00?00011??????????????????0??0???00?00???????200100????????????????????????????0?0?000000?00000000??????????????????????20?000?00000?01000000000000??0?01????0???????????????????????????00??

Austroraptor ?01??????????????????0???1100?0????011?0001??????????????????0?0001?0?0??????021??101????10111001111?11???????????????????????????????0?????????0?????????????????????????????11?1??0???001000??{1 2}1???10????0????0???????0?0?000??1??010????1?01???0?0???0001111??????0?1??????????????????????????????01????????1??1??????????????????1????????????????????????????????????????00?01?0???????01?0?0?????????????????????????????????0?00?0?????????????????????????????0?0?00100???0??000?000????????????????????????????????????????????????????????????????0?????????????????????0?

Adasaurus ?0110????0????2???????????????0????002??11?2?0?101??001??0?????0???0?1???0?0?0????????01100012?0111??0?111{0 1}100??011?1?1??????1?011111??????????????002211101110?200???1121100?111000000?00?110100100100?00??01100000?0???0?000???????????0?0????1????0????01111??1100????????????????????????????02?10111011000111210????1210?01?00??01??????1???0?????000??????0??0?1??1?00000????11???0000????????0?????????00??0???000?0????????00?001000{0 1}0?????????????200?0???0????????????????????????????????????????????00?000000000000000000000000?02??10?00???110000?00001?1????????0??????

Atrociraptor ???????????????????00?0??11100???????????????????????????????00?000???1????01000111001??????????????????????????????????????????????????????????????????????????????????????????????????????????????????????????0?????????0?00??00?10?3????00??????00?010??????????????????????????????????????????????????????????????????????????????????????????????????1???????????????????00?0??1????????????0???????????????????????????0??????????????????????????????????????????????????????????????????????????????????????????????????????????????????????????????????????????????????????

Achillobator ????????????????????????01110????????????????????????????????????????????????000101??????0?01200?111100??????0??011?11?????????0101????????????0??010220?0110100100?011010?00?21110?0????0?000?101????0??????1???0???????0?000??????013????0???????????10?00000??11?0???1???????????0????01?0????01010010?1??????12??????0010000?0??????????0??1?????0??0?01??????????1?1?0000000?0??0?????0??????????????????????????????????????????000??????????????????20000?0?00???????????????????????????????????????????00???0?0000?000???????00000??????????????????0???????0???????????????

Almas ?01??????0???11???11000??110000?1??20121?0011??0?0??????00?0?000?00??1??00000021??0100???????????????????????011???????????????????????????????????00??1??????0020?0??101?101???????00???????0??2???01??00??????0?0?????????2?0?011001000??1??1????0??1?10??????????????????????????????????????????1????????????????????1?1000????????0???3?????????????????0????????0010000000000?11?0?00????000000????00?000?000?????000000000??00???????????????????????????????????????????????????????????????????????????00?????00000?00?????????000??????????????????????01??????????????????

Archaeopteryx 1011000000{0 1}0?1111011010??111000111001110000000101010100?1110100000000001?0000020??0010001?1??10010??010?0?000021012112{0 1}0012??110101111000101100010001121??0001102000211021101?111100000?001000000{0 1}10?000000001100000?0?00010200101100100010100101100?00?001?111110101211301101111001000111010010202100111011011110010000010200121000000000100101?000000?001100100100110100000100001011000??000100?000000?001100?000??000?00??00???000000000000?0?????{1 2 3}??00020000?000000000?001000?00??00??0000100101??00000?0000000000?0?000000?00?0?0100001?0???000001010000010101?0101100?110000010

Alcmonavis ??????????????????????????????????????????????????????????????????????????????????????????????????????????????????????????????????????0{0 1}010?10?0100????????????????????????????????????????????????????0??{0 2}0???????????01?????????????????????????????????????????????11?01??11111010001111100102????????????????????0000??????????00???00??0?0??????????????????????????????????????????????01??????????????????????????????????????????????????????????????????????????????2?0??0?00000????01??101??01??0?1?00??????????????????????????????????0???????????0?????????????????{0 1}1111

Apsaravis ??????????????????????0??????????????????????????????????????0001??0??0???????????????1??11??10200??0???2?100021??22???0??1?01011031100001111300???000?1???1?2000???021?3220?12?010??1??1?12130100?00000??20??1?20?0???01????????????????????????????????????????010121?301?????10?0??????????????1??0??1?120???1?00????100??002??00????1??????1?????10??01???????0?????1???0?2??????????????02?10???????????????????0?1{0 1}??????0?????2001?{0 1}001??21??1??????00000?0?1001111?0111010{1 2}011110000101021110?{2 3}?2010011?20?1?001011110?101211211111?000?1?101????????01????0?1????????0???011

Anas 101??102?021002?2101001100?0?0?111?0002??00?1?100010?0???1?1020000100?021101?1????????101?111??21?0?111?3?11???10?2210121?1101101031111101111300???0012000?1?2?011?002103221?12?110001101?121311?0???01000211?1?20100???1??????????0???00??1???????1???????1????10?022??301???1?1???????????????????1??????2??00??0?0????10?00?????????0???0?????????????????1??????1??11110002121?????0???000121021111111122110211211111110110011111210110201112111121200101010?1{0 1}11?11110010001201000010111011111112314010111021?1111101111010211002111{2 3}221010201?10?0100000111?000??0100?10?001111

Beipiaosaurus ?0???????????????????????????????????????????????????????????????1????????????0100001????????1?????????????????????1??????????0???0?????????0?0010?100????????????????????????00?0????10?0?000?????0????????????0??????11???1????0?????????????????0????????????0???0????0100?0100010?01?0?100?0?1????110????0?1?????0?00????????1???0?0?0??0?0???????0?000?01010??00?????????????????????????????????????????????????????????????????????????????????????????????????????????????????????????????????????????????????????????????????0?????????????????????????????????????????0????

Buitreraptor ?0110??????????????00001011?000?????????000100??01?0?????????00??01???0??????021??100?0011111100111?010??0100011012?121???????10101111010?1?10?01???1?110100110220?0211?21120?11110?00??101100?1110000?0???0?1??00?0???00000000??1??0?000101?0?01?0?????0?01111?1010111120???????????????????????02110111?1?0???1?11?????12?11021??000?0??10?1?110??0?0000110?0???00??0????101000??0???00?00?01?00?0??????????0??????0?000101??0???000000000{0 1}0??????????000100?0?0?0??10?0?00100??0000100000001?100???????????0?000????00???00?????????0?00?00?010???????????01??0010??1100?000??010?

Byronosaurus ?????01???101?1100110?01011?00?????20120??????????1?100??????000001??11????00021??01?0?0???????010110????????0??02?????????????????????????????????????????????????????????????1????0?0?????????21??????????????0????10???0?{0 2}0?100?001100??100???10?001110???????????????????????????????????????????????????????1?????????????????????0??10?????0??????????00?0?1????00??0????0000?10????0????00000????????????0??????????????0??????001???????????????????????????????????????????????????????????????????????????????????000??????????????????????000100011?0??????1??????????????

Balaur ?????????????????????????????????????????????????????????????????????????????????????????????????1?11?1???10?0??0?????????0??1?011?11101011?1?000000??2?1?011110??00211121101????????0001??2110001000200??00?1???0?0???01???????????????????????????????????0?1??01?1?11?01??11100?00001?0000001??2??0???011001120?11??10122?000?000?10?00??0?011?????0??0?????????0????1?0?0?0??????????????01???????????????????????????????????????00000?????0??????????200???0?0??00?0?00000???00?00?????010?00???21??0?000020?100??000?????00???000000000?01?000????????01??????1????????0?0?100

Baptornis ???????????????????????????????????????????????????????????????????????????????????????????????21???????2?????????22?????????????????????????????????????????2??????????????????0??????????212?????????????????????????????????????????????????????????????????????????????????????????????????????????????2??????????????????????????????????????????????????????????????????2???????????????1{1 2}??????????????????11001??00??1???????2101000012????012?0???01?10?0??00?????????0???0?????????01?{0 1}1{0 1}?????????????20?11000011110211110021111?2120?2????????????????????????????????????

Bambiraptor ?0110?00100001201000000101110000?110022110?1000101?00011?0?0?000?000?1111000?01010100??1110?120011??100?0?1100?1011102????0001001011110?001?1000?0?10?21100101102000111021100?11110000000010001111?0000000000110000000?000?0000000?0013001?100101?00?0110001101100100111201001110001000010110010102010111?100001202100?0012?0101100000100000011110???0000001001?0100?11?100000?00?01?0?00?000010?0000?0??00000??000??000000001000??00000000??0??0?0?0?0?0002000000000?00?0?0?000??00000001?00010??????00000??00000?000?000000000000000000000?0?0100000011100001000011101100?100??001?

Coelurus ?????????????????????????????????????????????????????????????????00????????????0????1?0??0000100001?0000?????0?0002????????????0?0??0?10100????0??????????????????1?????11?0000000?00?00011000?0????0?00??00??0?0??0???00?????????????????????????10??????1{0 1}110??????111100??1?10??0000100???????????010101?0??????????11??1?0?????00??00??????00????00?000????????0????0?0?0????????????????01???????????????????????????????????????00100????????????????2???????????0?0?000000?00000000000010000???0????0??000????????00000000000000?000???????00??????????0????0??????????0?0?00?

Compsognathus ?0110???????????????010?011?000?0?0000???0011????000????0????0000000?0?00000001010101000?010?10?0???0?0?0???000?01?000001??????0?000000?10??????10?00?????????00100?100?11?0?????????00?001000000000001100?0????0?0???????00000000100100010100001?00??0?000?110001?1010010????1?0????????0?0?10???00101?1011000010000??0?00?00010?000000??1010?0?0??0000000100000??0??0000000000000010000?0??0100?000??0?0??0?0???0???0000??????0??0000?1000{0 1}0?0?????????0?20??0?0?00??????0?000???0???0??????????????000?????0?00??0?00000?????00??0??00?0000?000000????????0??000000????????0?0?000

Caudipteryx 00110?????????????0?110?010?00011??012210001?00?0????????????21?1000???0?10021???????00????0?1000???????0???01????210?111000??00?010001?00??100020010020???11?02200?111?10?10?11?01??0???0100000100000000000?1102000????00?0??1000101100010100111?1010??0?0?1110?110001110100?1100000001?0001101?01010111011000010000???010100010000000000100101?0??0001000?110100?010??10?000000000??0?0??0?01000000???????????0?????0000????000??00200?000?0?0???????????20??0?0?00?0??????0?0???0?00????????????1??0000???00000?00000000?0?0?00????000000?0??20??0????????01000??01?110???0?000000

Chirostenotes ???????01?01101??0???1?110????????????????????????01?10????0?2111000000201???1???????????????1101?111???1?12???0????????????????101????????????0101?0021?00?010220001110?0?10?01???00?????100000200000??000??1??20?0?00??????????????????????????12???????1?1???????0??????00??????00001?0?1001000?01?1?101?0000100?0001110?0001?000000??0??0????????01????????????0?1??1?00000??????????0?0????10???????001????00???????????0?10?????001??00??????????????20000?0000?????????????????????????????????????????0000?00??000000?0????????00000????00??001010?00??0??????1???????0?0???0

Citipati ?01101001001??2210001111110101011?101221000110000100110001112211100110010111?1????????1011001100101111001??201??002000111?0111210110000100?1100001110021100111022000?11010?10?210100000000?0000010000000100001102000000000?0??101011110101011101012010????00101011100101101001110000000000000010001010111011000010000110010100010000000100?0010111110111000111010000?1021000000000011?0?00000012100000001102?10?0000?000001000010??002001000{0 1}0?00?00100001020??????0??00?0?000000?00000000000?00100?0?00?0???00000??0?000000000000000000000002?0000000001000001000{0 1}10?011011010?0??00

Conchoraptor ?0110?????????1???00111?11???1011??01221000110?00????1?0?????21110010?0?0111?1?????????01010110010??1?012?110??10????01111??1?21011000010???????011?00210001010020?0?1101001??11010000??0010000000?000???00?01102?0?00000??0???0101111?101011101012????????1????1????1?10???011?0???0?000000111000??1??1??110??????00?0001010000?????0???0?00?0??011011?00??1101?000???21000000000001????0?00?????00000000????????0???0100100??????00?0?00????????????????????????????????????????????????????????????0?????????00?00??00000000000000000000?02?01????0?010?000?0002?00??????0???0???0

Confuciusornis 10110?????????????11010?01??000?1??0??2?00?110?0{0 1}??01???????00001000?10?0001?1????????0???1??1??0101110?2???0?2????2??1110110100?1311140001111000010112101?0??100?0012103210112?110??11?120211010010?000000001102000???01??0??0011?0??00010100?01?0110????11111?10101211201101111000010120110010002??0111?1101112000101101000011?000000000?00?01?0??00?1001?1101???011011?100?1001001??0?????011{0 1}0010?????????10??0??011200??0{1 2}0111000001000010010001200?00?0000?0?????100?00211??000000000010102101011{0 1}?10?0000101000000000101001000110001100?00?000???1000001010?00??1100?110001111

Cathayornis ?0????????????????1?010??????0?1?????????0???????????????????00?0??????????00001??????0????????2????1???2?????2????2???0??11?0201031110?011?1300?0?01111?0?00?100??01?103122????1????1?????21{0 1}1?0?100??0???0????0??1????1?????0??1?????????1??????0?????????????10?012??30????2?1?????????????????????????????11??0?1?????0?000????????0?????????????????????1??????????1?0?0????????????????02{0 1}0?01??????????????????????????????????011??0010?20??1??110?101????01?000010110?0102011110?{0 1}?1?????210120?11?0?101011?0?0000???1?0?0?1{0 1}10??????????110????????0??????01???????????????

Concornis ?????????????????????????????????????????????????????????????????????????????????????????????????00?1??????????????????0??11?02?1031110?0?1???00?0????????????1010???11?322??0???1???1????1211??0?1000?0???0????????????1???????????????????????????????????????1???12??3?????????????????????????2?1????????111????1????1?2??0?????????0?????????????????1?????????????1?0?0????????????????0{1 2}???????????????????????????????????????011???010?20??1?01100101?0?001000001?110?0102????10???1{0 1}1?{1 2}????????????????????????00????0?100??100??1?1??0?110??????????????????1100110???11?1

Chauna 001??012?0210002?101010100?0?0?111?00021?00010100010100??1?10000000??0021101?1????????1011101??2100?111?3?11???1012212111?1000001031111101111310???0012101?1?2?010??02103222?12?010001101??21301001000000020101?201000??1??????????0???00??1??????11???????1????11?012??30????2?1???????????????????1??????2??????0?0????10?00?????????0???0?????????????????1??????1???1110002121?01??0???0002110211{1 2}111102211011121111111011001111121011020111211112010010101111111?11110010001201000010111010111112314010111021?11111111110202120021112222010001000101000101110000?11101?11?0????1

Crypturellus 10???012?020002?2001010100?0?0?111?2002100001011100010???1?10000100??102?001?1????????1110111??2100?01103?110??1??22??121?1101001031101101111300???001210001?20211?002103222?12?0100010010121301?0???0100020001?200002??1????????1?0????0??????????1???????1????10?022??30????2?1???????????????????1??????2??????0?0????10?000????????0???0?????????????????1??????1???1110002120?01??0???0002211211{0 1}010001111101110011011111111001021011121121211112?10002101101011?11111010001201000011101010111110314010111020?12011011110202110021112221010101?10101000001110000??1100?10?0?0???

Crax 101101?2?021000??001010100?0?0?111?20021000011100000100??1?10000000000021101?1????????1000111??2100?011?3?120??1??22??121?1100201031112101111300???0002100?1?2?011?002103220?12?010001101?1213010010001000201?1?201002??1????????1?0???00?????????11???????1????10?022??30????2?1?????????????????2????????2??????0?0????10?00?????????0???0????????????????01??????1??111100?2121?01??0???00022102112111112211011111111111011001101121011120111211112110002101111111?11111010001201000010111010111111314010121021?11111111110202110021113222011001000?01000001110000?1110001??0?????

Daspletosaurus ?1100?0000000100??0000010101?0000?0110120102110000010001000000000000?100000000001010110???000???001?10??0????000??0????????????000000010000???01?00103100010000010?1011010000?0100?0000??01000002000000010000000000000??0???00?000010101100000000?000?1000???????1?100??00??????0???????????????????1??????0??????0??????00?000????????0???2?????0??0??0???00000??????00100000?000011?1111111100000000???0000000000???00001?1?000??00000?00??0?0?????????0020010?0000101?0?000000?00000000000010??0?????000??00?00?0000000010000000000000000?0??00??02000??000?000100000110?000??????

Dilong ?0100?0000000?100100010??10100000?01101000010?0000?0000??????000000??10?000000001010111000??010100??0?0??????0??01??0?0?1???????000001?000????0010000001??10??01101?0?0?01000000000?00??0010?00100?000000000?0?00000?0??0?0?000000010101000000000?0???11000?1100?101011?1??00?0000010001000000001?101010101100001000000100000001?00??0?0?0000100?0???000000000000??00?0100000000000110?011??00?00000?????0000?0?00???000001?10000??00?00?00????0???????????20?00?0?00000?0?0?000???00000000??0?0??0????1000??00000???0?00000000000000000000??????0000?0?10000??0000000????????0?{0 1}???0

Deinocheirus ?????????????????????????????????????????????????????????????????????????????????????????????????????????????????????????????????1?00000100???10000???????????????????????????????????????????????????????0??????0?0???0??????????????????????????????????????????00??10?0??111000010000000000001????????????????????1100??????????00??011??1?0??????????????????????????????????????????????????????????????????????????????????????????????????????????????????????????????0????????????????????????????????0?????????????????????????????????????????????????0???????????????00000

Deinonychus ?1110???1???????11?0000?0111000011100221111?00?1??000011101000000000111?1000?010101000?11000120011111011????00110110121?10???1?0101110010011100000100221110101002000111120100111110000000010001101001000000001100000?0?00?0000??00?0013000?00????000?1?10001001??11001111010011100010000101100102010101100110011212100?001210000100000100000010110000000000100000100?110?0000000000110100??0001000000?0????000???????????????0000??00000000?{0 1}0?0???????????20000?0000000?0?000000?000000010000101?0???00000??10000?000?00000000000000000000000?010000????????01?000111????????0?00000

Dromaeosaurus ?0??01000000000010??0?0?0???0??0111????1111????101?1001100??0000000011111000?000101001?????????????????????????????????????????????????????????????????1??????????????????????????????????????11?1?0????00?????00?0?000???0?00?000??0??0?0?00?000000010000??????????????????????????????????????????????????????1121???????????????????0??00?????000???0???10000?????????0?????00?01101???0??????0??0000?00000??000??000000000000???????????????????????????????????????????????????????????????????????00?????????????????????????????????0???0????0101110000?0000?0????????????????

Eotyrannus ????????????????????00??0????0?????100???????????0???????????00??00????????00000101001???00??1?1???????????????????????????????0000000100?0????0?0??????????????????????????????????????????00??????0????000????00?0???00???00??00?10??10???0??????1?11100?0??????00??11?0??0??1?0?0??0???0?00??????????????????????????1????0?1??000??0?00?0???00????????????????????????0????00?0????1?????1?0000???????????????0??0?000000??00????00?????????????????????0000?0????00?????000???00000?????01?????????00???????????????????????????????????????????????????????????????????????0???

Erlikosaurus ?0110?0210?1?0??10100111011?10001?100001000110000?00100??1111210010000020001?00100001?????????????????????????????????????????????????0?????????????????????????????????????????????????????0?00?021????00??1??00?0?00011?1?11?0001001?0010100001010000001????????????0?????????0???????????????????????0?0?001110001??????????0??111100???0?????0100??0????0101?????1?0?0?????00010110?000??0?00000000001???10?010???00000000000??00????????????????????????????????????0?000000?00000?000????????????????????????????????????????????0???000??0?000?0????000??000?0????????????????

Erliansaurus ????????????????????????????????????????????????????????????????????????????????????????????????1???0???????????0????????????????????00?10????00010????1???1?????????????????0??0??000?00?????????????????0????????????11??????????????????????????????????????????0?10010?0010100001010001100001?????01?????????????0100????????1?00???00??0?1?????0???0?0??????????????????????????????????????????????????????????????????????????????????????????????????????????????????0??????????????????????????????????????????????????????????????????????????????????????????????????00??0

Elmisaurus ???????????????????????????????????????????????????????????????????????????????????????????????????????????????????????????????????????????????01?1?????????????????????????????????????????100010??01????0?????????????????????????????????????????????????????????????????0??????00001000?0??00????????01?0?0010000??11??????1??0??00??0??1???1??????????????????1?1????????????????????????????????????????????????????????????????????????????????????????????????????????????????????????????????????????0?????????????????????????????????????????????????????????????????0???0

Eosinopteryx ?011???????????????10?0??????0??????????????????1????????????000??1????????00020???1??0??????????????????????01??1?10?0?1?2???0??01??1????????0010?001?1??00??022???2?10???0?????????????????0??00???0?0???0????0???????????2001?010????0?????????0???0??0??????0????1?020?0??1100????1100?111???0???01?1???0???????000?010?0?02100????00?1??1???????????00?????????1??????????????????????0?020??000?????????0?????????????????????0????????0?0??????????02?0???0?0??0??????0????????????????????????00??????0?00??0??????????????????0?????????00?0???????????????????1??????01???0

Falcarius ?01??1000??01?0121??????0???1???????????00????????00000??????1???00??????????00110000????10?01101?101?000?110011102?011?????????00?0000110?1?000{0 1}00100?11001010220001?1?10100000001000100010000000?00000??0??10?00?0?011111111???????????????????00?????001?11??01????11?01?010100010001000000100021101???0?0?0?10?000010???000000000000?0??0?0???????0?000?????0?00?1???????????????????????????????????????????????????????????????????????????????????????????????????????0????????????????????????????????0?????0?????????????????0??????????????????????????????????????0??00000

Guanlong ?0110??0??????????00011??11100000?00111?0??20{0 1}000?0?000??????0?00000??0?000000001?10?10????0??0?0???0?0?00000?????0???100?0??????000?00?1?0?11000001030000100000101?000?10?00000001000??010000??0000?000100??0?00?0??01001?000000001000100000000?00?0011?00?110??1?10111101000110001?00000000000101?00101?110???1000?00000000001?000000010020??0?0??00000?0100000????00?0????????????0???????????????????????????????????????????????????????????????????????????????????????0????????????????????????????????0?????0???????????????????????????????????????????0???????????????00000

Gorgosaurus ?1000?00?00000?0?10000110111000000011012010211000001000?00?00000000001001000000010101101000000?0001010000001000000000000000001000000001010000?011001031000100000101101101000000100?000010010000020000000100000000000?010010000000001010110000000000101100000000001010000000?01000101000??000000??00010001011000010000???00010001000000001002010000000000000000000?01?00010000000000110?11111110000000000?0000000000???00001???000??00000?00??0?0?????????0020010?0000101?0?000000?00000000000010??0?????000??00?00?0000000010000000000000000?0??00??02000??000?000100000110?000?0????

Gallimimus ?0111?11011010101021001101111012110000000000000010000001?0101000000001020001?1????????0011?011000010?0001?100000000000100??????0112000201000??10100100000011000110010000001000000011000100100000203?0000011111012111001000?0??110010012001010010100?00????1?1100?1{0 1}0011010001110000200000000110020101010111?0???000001100001000200000?0011?0110010000000000?00100?01?0000000000000001??010?001100?000{0 1}001000000?000???00000001200??000001000{0 1}0?0???????????20?00?0000001?0?000000?000000?0?000?0???1??00000??00020?000?0000000000000000?000?00?010??1002??0000?000000?0???????0?00000

Garudimimus ?0110???01101????02100?101011000??000000000000?01?0?0001??1?00?0000000020001?1?????????01100???000??0?001??0???????????????????????????????????????10000001100??????????00?000000010000?001000001000000?01??110?211?001????0??110010012001010010101000????11110?????????0????????????????????????0???01010110010???00????00100?1000??000???0????00000000000?0000???0?000?0?0000000000???00?00??00?000?001000000?000??000001011000??000001000???0???????????????????????????????????????????????????????0????????00?000000000000000100000000003?010?0000???000??0001000????????0??????

Graciliraptor ?????????????????????????????????????????????????????????????????????????????0{1 2}0??1??????????????????????????0???11?12????????????????01001?1100001??????????????????????????????????0??0010??11?1??01?0??00????????????0???0??????????????????????????1?????????????110?01??11110?0?010100?0?001?????????1?????2011???00?????????000???00??0????????????????????????????????????????0???????01??????????????????????????????????????????????????????????????????????????????0?0???00??0???????????10?0000??0?00??????????????????????????????????0?????????????????????????????00??0

Gobipteryx ?0????????????????1?01010?0??0????????????????????????????11?000?0?????????1?1????????????????????????????????????????????????20???????????????????????????????????????????????????????????11????????2???????????????????????????1????????????????01????????????10?01??????????????????????????????????????2?????????????????????????????????????????????????0????????????????10???????????????110010?00110?10????00?????0???0?0?????????????10?????????1??10000?0?1000001?1?110???0???????????1?121??{0 1 2}0??????0?1????????000???10?00111000?1?1?0?????????????????????????????????????

Gallus 101??102?021002?2000010100?0?0?111?20021?0001?1010001????1?10010000000021101?1????????101{0 1}111??21?0?01003?120??1??22??1{1 2}1?1101201031102001111300???001211111?2?011?002103222?12?010001101??21301001000100020100?201002??1?????1??1?0???00?1??????111???????1????10?022??301???2?1?????????????????2????????2?1?0??0?0????10?00?????????0???0??????????????1??1??????1??111100?2121?01?00???00021102112111112211011111111111011001101121011120111211112110002101111111?11111010001201000010111010111111314010021021?121111111102021100211132220111?100010100000111?000??0010110?000111

Huaxiagnathus ?01?0?????????????0001???1??0?0????00??0?0???????????????????00?000??????0?000101010?00??0???1????????0??????00????00000102???000000000?10??010010000020?0??0000100?000011?0??0???0??0??001000000000?011??00????00?0??????00000000100100010100001?0???1??00011?00100010010000?1000010001?0000000?000101?10110000100?0001000?00010000000000?00000?0????00000?00000??0??0?0??000?000??10???????01???000?????????00???????????????????0000?1?0?{0 1}0?0????????00020??0?0?00?0??????0?0???0??????????????????010????00?00?0???0000?????000?0??0??0??????0000????????00??00000?0?00??00?0???0

Harpymimus ?011??????????????21000????000?????0000??00??????????????????00000?0000????1?120??1?0?0???00?110001?00001?10000?00000?1????????0?0?0?0201001000020010??00011?????????00????00?????????????00000010??000001010???1?00???00?1?2011001001?0010?00101?10?0??000?110??1?0??1000000120000100000000010020???0???01?0???10000010000?00?1?0000?0000??110?00?0?000????00100??0?0?0???00?0000??1??????0?1100000????????????0?????????????000???0000?000{0 1}0????????????????????????01?0?000000?00000000000???1?010?000000?00000?0?000000????0000000000000?0??000?1????????0????000???????????000?0

Haplocheirus ?0111?000010?????011011?01111?001??00?01?0011?000??0?0??0????00000101101?000?01110?0?00???0??0?0111101??0?1000?00???0?0011???0?0?02000??210?0100210?00?????0??0??000?1??01100?10000000?0?0?0000000??00011?0??11001000?100?00100100000010010100001??001??000????1??0100101010000001010001000001001?10101110110???100000000000?00100000?0000101?1000000000000000000???0?01000000?000000??01?????1000000?????????0???????????10????0??00000?00??????????????????00?0?0??00?????1?????00?00??????????????0??0?0?00000??00?0000??00?00??????00?0?????000?????????????0??01???????????0???0

Hagryphus ????????????????????????????????????????????????????????????????????????????????????????????????????????????????????????????????????????????1000001???????????????????????????????????????????????????????0???????????????????????????????????????????????????????????????0?010100000001001?00100????????????????????0?11????????????????0??0?0???????????????????????????????????????????????????????????????????????????????????????????????????????????????????????????????????????????????????????????????0?????????????????????????????????????????????????????????????????0???0

Heyuannia ??11???????????????????????????????????????????????????????????110011?0??1????????????1???????????????0?2???????????0?111?????11?110?00?10??1?0?01010021???1??0220??011?1??1??1?010000?0?????0000000?001??00?1??2?00???????0??1???????????????????2?1?????00?11011100?01?01000110000001??00?01???01000111?1?0?0011000???010?0?00?0000001?1??0?01?0???11?000????????0?????????????????????????????????????????????????????????????????????????????????????????????????????????0????????????????????????????????0?????????????????????????????????????????????????????????????0???0?00?

Hesperonychus ??????????????????????????????????????????????????????????????????????????????????????????????????????????????????????????????????????????????????????21??010???????????21121????????????????????1???????????1???????????????????????????????????????????????????????????????????????????????????????1????????????1????????1????????????????????????????????????????????????1????????????????????????????????????????????????????????????????????????????????????????????????????????????????????????????????????????????????????????????????????????????????0???????????????????????

Hongshanornis 101???????????????11010??????0?11??0????00???????????????????000001??00????1?1?1???0??0??01????0????????2??????????2???1??11?0101031110001111300?0?0112???01??0??????2??32221?2??100?1?????21{2 3}01000000000?00?01?1?????????????0??1?0????0??1????????????0??1????10?012??3?1???1?1??0???????????????????????2?11???0??????????0??????????0??0?????????????????1??????????????0??0???????0???0?01???01??????????????????????????0????1????0????110?????????0??10???0?1??1111???10???2???0????????????1??2000??0010?0??0?0??00???????1???100??{0 1}?0??2??????????????????????0100?10??0???1

Hesperornis ?0????????????????00010100?0?0?????00021?001????00???00????1?000001???1?0001?021??00??10101110?2100?011?20100?210?22??11??10??0?103111?????????????0?02100?1?2?00????2103220?12?0?0001??1?1213110010?10?00??1?1?0000?0?????????????0????0??1??????01???????1????0??0???????????????????????????????????????2?????????????00?0?0??????????????????????????????1?????????1??100?21111??????1?0?1110?111200110?1010??110011100??0?0???102101000012?0?0001000?0???10?0??001????????0???0???0????????????????????????20?110000111102111100211112212002??00??????000?0?0?000?0100?1???0????

Incisivosaurus ?0110?01?001??1??1000101011100001?00122000011000001011010111121010001001?1?02020??0002???????????????????????????????????????????????????????????????????????????????????????????????????????????????????0??0??01?0??00???0020?00011110001010010111010?001?????????????????????????????????????????????????????????????????????????????0??10?????0110??1???1?0?1?0????00?1?0???00001010001?????000000000100001??0000??0000101?100??00????????????????????????????????????????????????????????????????????????????????????????????????????????????????01010000??0000?0?0??????????????

'IGM100/44' ?????012???????????????????????????????????????????0??????????????1??11???????1????????0????????????????????????????????????????????????????1000000???????????????????????????????????????????01?1?001???00?????0?0??1??????3????????????????????1???0????????????????????1?0111000?000???00000???????????1???0?0121????0?????????0???0?????0?0?????????000???????????????????0???????????????????????????????????????????1?1?????????????????????????????????????????????????????????????????????????????????????????????????????????????????????????????????????????0??????????????

'IGM100/1126' ?0???002?0000?11{1 2}01?010??100?0?011?20221000110?01010100??0??00000000?11?00000021??00?0??????????????????0????01102???????????????????????0??1000?01???211?010?0020?0?01012101??111000??????2?001210001?0000011?00?0?02?????????????0???00??1????????????1????????????????0??????0???????????????????1????????????????????102000????????????3?????????????????0????????0010000000001111?0?000???00000?????00??00?000??0??000000?00???0???????0??????????????????????????????????????????????????0?00???0000???10000?00??0??000?00?????00??000?0?02?000010100111?0101?001??????????????

Iaceornis ??????????????????????????????????????????????????????????????????????????????????????????????????????????????????????????1101?110311??????113?0?????????????2????????????????2?01??1??????21?????????????20??1??0???????????????????????????????????????????????0?02?????????1?????????????????????????????????????????????????????????????????????????????????????????????????????????????????????????????????????????????????????????????????21?02211?1101010?111011????????????????????????????111314011111020010??1??1110212110021????????????????????????????????0100?1???????1

Ichthyornis ?????0????????1220?????????????????????200111????0???????????0000??????1000??021??10???1?0111??2100?1???2?000?21???2???01?100?001031110?01111300???0102?1??102?00????210322??12?010011??1?1213110???00?0??20????00??????1?????????????????1???????01????0??1????11?022??30????1?1??????????????????????????2?????????????10?00?????????0??????????????????????????????????1???2?????????????002{1 2}0?11??????????10{0 1}111101110100120???1?20011?101{1 2}?2110220100001010?11101111100121011100000101{0 1}101121111231201101112001000101111021111002111{1 2}2?10002?1?100?10???0101??00?00100?10??01111

Jinfengopteryx ?011????????????????01???11100??????????????????1????????????00????????????00020??01?????????????????????????02?0?210200112??????01111??0???1000?0?00??1????????????2??????00????1???0???????????????0?1???0????00????????0?20010010010?010100?01?????0??0??11?1?11111?11010??1100?1??010000001020???01?1????????????01?01??????1??0???0001????1?0??0??0??0100?0????1?0????????000???1?0?????01?0?00????????????????????????????????00????0??0?0????????????00?0???00?????????????????????????????????0??????????????????????????????????????????00??????????????01????????????0????0

Khaan ?0110?????????1?10001111110101011?001221000110000?00110??????2111001?0020111??????????10110??1001011110??0?2?1??00210?111001??110110000?00?11000011100211001110220001?1?10{0 1}10?11010000??0010000000000000100001102000?00000?0??101011110101011101012?10????00111011100101101001110000000000000110001010111011000010000100010?00000000000100?00101?0?101?1000111010000?1021000000000??1???0?00001010000?????????0????0??????1??0010??00200?00??0?0??00??0000020????0?0??10?????000???0???0???????????0??00????0?0?00?00?00?00????????????0??00????00000???????????0????0?11010010?0?0?0

Limenavis ????????????????????????????????????????????????????????????????????????????????????????????????????????????????????????????????????????011?13????????????????????????????????????????????????????????????20????????????1?????????????????????????????????????????????????????1?????????????????????????????????????????????????????????????????????????????????????????????????????????????????????????????????????????????????????????????????????????????????????????????????????0000{0 1}010101011?11{1 2}313011?11???????????????????????????????????????????????????????????????????1??

Liaoningornis ??????????????????????????????????????????????????????????????????????????????????????????????????????????????????????????????????3??1??0????????????????????????????????????????100?1???0?21211001000?????0???????????????????????????????????????????????????????????????????????????????????????????????2??1???0????????????????????????????????????????????????????????????????????0???????????????????????????????????????????????????????011?01????????0???0?????????????????????0?????01???2???????????????????????0?1?0??0011111020100?00????????????????????????????????????

Lithornis 101???????????????01010100?0?0?111?2?02?00?0???????????????1000010????02?101?1????????1????????2????????3?1????????2???{1 2}??11000110311110011?1300???000210101?20010???2103????12?01000???1?12130100?????0??20?01?2010????1????????1?0???00??????????1????????????10?022??30????{1 2}?1??????????????????????????2??????0??????10??0?????????0???0??????????????1??1??????1???1?100?212??????0???0002?1121100?0001111101110?11{0 1}1101?111?0102101?0111{1 2}12111221100?0101101110111110010001{1 2}1100001010101011111131301101102{0 1}012011011110201100021112221010{0 1}?1?1????????01?1??00?????????????111

Mahakala ??????????????101??????????????????????1?00???????0?001100???????????????????02???1???00??0?11?011?1110?1?100011012?10????????????????00??1110000?100?211101?2???????????????0111100????0011001101?00000??00?11?0??????????0??????????????????1??1?????????11??????0?0?1101??11??0?000???0?0?11???????1?201??????11?0?10????00?1000?????0???01??10????0?0?0???????0??1?????0??0????0???????0??{1 2}?????0????????????????0??1?000???????0000100000?????????????????????????0???0?000???0????????????100????0??0??00000??0??00?????0000000000000000?010000????1???0???011?0????????0?0???0

Mei ?0???????0????????11010?????00?01?0?012100001?10?0??100??????000?010?11?0000?021??01?001101111001??1010?01???011022?1110112???101?111{0 1}1?0?1?1000000?012111?1??0020??2110?110?0111100?0101010000{0 1}11101100?000?0?00000???01??030110110???001?100101?0????0?01111??1110111110?0??1000?00??00?00000?00??1?1?101110001?110000010?01?2100000000010010110???000000??010011??1??10000??00000?1?000?000100?00?????????00?0?0??00??0101???????00001000?0?0????????00020000?0000?00?0?000000?000000?000?01?1?0???00000???0000?000000000??0000000000?00000?020??0???100??110101100?1100?110?00000

Mononykus ?????00???????112???????????????????????????????????100???????????????????????2???????????01?1?111010011????10?2??????1???1000?000200030210?12201100??1????1?2000???????3??0?021010011101211100030?00001??00??1??0?0???01??010??????0?????????????????????01111???100000000?011001000????00?1?????????11??1??00010000????0??00???001100?1???1?00?????00?000?00?01?01?0??1?100?0??????????????1{1 2}????????????????????????1??????????????001???????220???00???2???0?0?00?010??002000?00?0000?000??0000???300?0?????0??00??????000100000000?0000?0?00??000???????0?0???0?0????????1?001??

Microvenator ?????????????????????????????????????????????????????????????2??100????????????????????011?0?11000111100???0???1002?????????????0000??01000?????{1 2}??1002???0111????0?????10110?0101100?000010???????0??0???0??11??0?0???00??0??????????????????????10??????0?111?????011110??0??????????????0?0???0???011????????????????????0????00000?1????0????????0?1???????10?0?????????????????????????????????????????????????????????????????????????????????????????????????????????????????????????????????????????????????????????????????????????????????????????????????????????????000??

Microraptor 101????????????????0010????110???????2???????????1????????????0?000?0?1????0?0{1 2}00000?????001?1??01?1000?01???0110?1112111?01?1001111110100?111001011012111?001002?0?211021120?11111??00?001110111110?000??00?11?000?????000?{2 3}0?100?0??3001?1001?1?0?0?11000?11111?1001112110??111100010111110010002111101?100001201110000121?1121000001000100101?0??000?0011001001001?101?011100??1?10??0??0?01?0?00???????????????????????????0???0?000100?{0 1}0?00?0000?0000200???0?00?00?0???0100?00??1?0?000?????????000?????0?00?00000000????000?00??0000000??11??00?0110??0101?01?1?1100?100100?10

Nanshiungosaurus ???????????????????????????????????????????????????????????????????????????????????????????????0001?00??0???0??????????????????????????????????????2?0???0???20020?0?01?2??????????????????????????????????????????????????2??????????????????????????????1?010??????????????????????????????????1201?????????????????????0???0??1???1???????????????0??00??????0???????1????????????????????????????????????????????????????????????????????????????????????????????????????????????????????????????????????????????????????????????????????????????????????????????????????????????

Neimongosaurus ?????????????????????????????????????????????????????????????2????0???????????0?000?1?1??10011?0101?0?0?1??2?01000210?1???????0001000000??????????????21100??2???????????????00?00000??????000000?20100??????1???0?0???11?121????0??????????????????????0111110001??010?1?????????????????????????????01000?00??1000????????0??001110100?????0?01????00?000?????0?00?????????????????????????????????????????????????????????????????????????????????????????????????????????????????????????????????????????????????????????????????????????????????????????????????????????0???0???

Nothronychus ?????10?????????20????????????????????????????????00100??????????????????????001000??????00001101??100001??????1???1???????????0?0?0??01100?????????????1?????0021?0?11??????????0???010??????0???????????0?????0?????01111?1????????????????????1?????0??101?0????0??00????????????????????000????01?0?????????????1????11???1????00???????0????????0????0???????0??????????????????????????????????????????????????????????????????????????????????????????????????????????0???????????????????????????????????????????????????????0???????????????????????????????????????????000?

Neuquenornis ??????12????????20?????????????????????????00?????00????????????????????????????????????????????00??0???????????????????1?11011?1031110?01111000????????????????????????????????110??????????{0 1}010010?20????0?????0?00???????????????????????????????????????????1????2??30????????????????????????????????????????????????????????????????????????????????1??????????????????????????????0???02???????????????????????????????????????011???????21?????110??0100?001000??1???1?0???0????{0 1}?????????2???20?1??0??????????????0?1????????10???101????000???10000010???????????????????1?

Ornitholestes ?0110??0?0?00?1???0?010?011100001?00000?00?1000001?01011????00000000000?000000?010100????00011?0101100000?1000?0010??1???????????????01?000??????0000001??010?0010?000101?10000001??0???????00000????00000?010?00?0?00?0010000100010010001?1000?1000000?0001110??????11010?0???????00????000100??00010?0101?0???????0????001000100000??0??1201??00000000000000000?00?10000?000?000?01??000?0?01000000?0??000000?0?0???0000000000???000001000{0 1}0?????????????????????????0?0?000000?00000000000??0??0????1000???0?00?000?00000000????????0000?00?0000?????0?000?0?0001?0????????0??0?0?

Ornithomimus ?0111?110?101?10102100010101101211000000000000001000000??????0000000010?1001?1??????0?001??011000010000010100000000?001001?????01120002010000?10100100000011000110010000001000000011000100100000203?000011111101211100?00??0??1101100120010100101?1?0?????101100?1???11010?01?10011200010000220020101010111?0???000001100001000200000?0011?01100??000000000000100?01?0000000000000001???10?00110?0000?????????0?0?0???0000000??????000001000{0 1}0?0????????????0??0?0?00??1?0?00000???000000??00??????1??00000??00020?000?00000000000000000??0????0??0?1???????0??00000000???????0?0????

Oviraptor ?0110?????01?11??00??1??111101011??01221?0?110??0100???0?11??2111001100?01?1?1????????0??1???1??1???1?0?????0???????????1????121?1?0?000001?10?000100?21?00?????????????????????????0????????0??0?????0010000???2?00?000???????01?1?0100?1?1?00?012?10????0?111?11100?11?01??11100000000000000100????????????????????1000??????????00??100?00?01?0?101?1?001110100????0???0????0000?0?????1??01???0?0????????10?0?0??0??00?00??1???0??00?00????0????????010?0?00?0?0?????????100???0???????00?????????000?0??000??????????????????????????????????0?????10?????0?02?0??1101101??0???0

Ostromia ?????????????????????????????????????????????????????????????????????????????????????????????????????????????????????????1??????????????0???1?0010????????????????0??????112?????1?0??10?????????01??0?0??0?????????????????????????????????????????????????1??????????1?0?????1????0????1?10010?????0????1?0???1???0?????????????0???0?????0?????????????1???????????????0??????????????????0??????????????????????????????????????????????????????????????????????????????????????????????????????????0????????????????00??0????????????????????0?0???????????????????????????1???0

Proceratosaurus ??1?0??10?00?1????0001010111?0000?0??????????{0 1}0?00???????????000?000?00??0000000101011??????????????????????????????????????????????????????????????????????????????????????????????????????????????????00??????0?0??????????????0?0???0???0??????0?????0??????????????????????????????????????????????????????????????????????????????????2???????????????1?0????????01?0?????0000?10??0??????00?00???0??????00??????????0????0???00???????????????????????????????????????????????????????????????????????????????????????????????????????????????????????????0????????????????????

Pelecanimimus ?01??1???1????1?2?21000??10010??????000?00??????1???000??????000?000?00??0?00021??0001???10001?00???00???????????????????????0???02???2?10??0?10200????????????????????????????????????????????????????001?1????0?1???????1?10?100100020010100101?0??00?10001?????????10?00?111001120000000012002????????????????????1100???0?0????????011101?0??0?000?0????0000??????0?????????0?0?1???????011???0???????????0?0???????????????????????????????????????????0????0?0???????????????????????????????1??0000???000??????????????????????????????????0??????????????0??0???????????0???0

Parvicursor ??????????????????????????????????????????????????????????????????????????????????????????????????110???????1??2??????????????????????????????????????1?1??????0????0???3??0??110100011?1211000020??00???????1??????????????????????????????????????????????111???????????????????????????????????1??011211?0???10000????00??002??????0?????????1?????0????????????1?????????????????????????????????????????????????????????????????????????????????????????????????????????????????????????????????????????????????????????????????????????????????????????????????????????????????

Patagonykus ????????????????????????????????????????????????????????????????????????????????????????????1?011111?????12?10?2????????????????00200?30210?10??110???1????1?20???00????2?101?1100?00010101110??0????001??0??1???00????010??????????????????????????????????1?1??????0??000?0???????0????00?1???????1??1??1????0??????????????0?????1???????1?????????0???0???????00?0??1?0?00????????????????????????????????????????????????????????001??????????????????20?00?00?01?0?0?00???0?00??????????????????0?????????0???0????0100000000000?00????????0???????????????????0??????????0??0?

Protarchaeopteryx ????????????????????0????????????????????????????????????????????00????????02020??0002????00?1???????????????01????10?????0???0???1?????00??1?0010000??1???????2200?1?1????00?????0??????????0???000?000???0????1?0????0??1?101??0???????1??0???????????010?11?00?1001111000??110??10?0100100000001010111?1?000010??00000???000100?000000010010?1??????1000?????0???1??????????????????????????????????????????????????????????????????????????????????????????????????????????????????????????????????????????????????????????????????????????????????????????????????????????0????0

Pyroraptor ???????????????????????????????????????????????????????????????????????????????????????????????????????????????1????????????????????????001??????????????????????????????????????????????????01??1?????????0?????????????????????????????????????????????????????????????0????????????????????????????????????????1???????????????????????????????????????????????????????????0??????????????????????????????????????????????????????????????????????????????????????????????????????????????010??0???????????????????????????????????????????????????????????????????????????????00?

Pedopenna ?????????????????????????????????????????????????????????????????????????????????????????????????????????????????????????????????????????????????????????????????????????????????????????0?0?0??0110??????????????????????????????????????????????????????????????????????????????????????????????????????11000120??0?????????????0??00?????????????????????????????1??????????????????????????????????????????????????????????????????????????????????????????????????????????????????????????????????????????????????????????????????0?000????0??00????????0????????????????01?????

Pengornis ?0110?????????????10010??0?0?0?????0?22??0??????0?????0??????000?01????????0?0?0??00???01??????20???1???2??????????2????1?????211031110?0??????0???001?1????????????????3??2?????????1?????211???0?002??0??00???0??0????1?????0??1?0???00??1??????0?????0??1????10??12??30????2?????????????????????????????????????1???????0?????????0????0????????0?????1??0?????????????00?1000???1?0????00200?00???????????????????????????0???0??001??00100????????{0 1}00?00?????????111{0 1}0?1101000{0 1}{0 1}1?????1??1???10{1 2}{1 2}0??1???1??????0???000????0??0{0 1}?100??1?1??0?100????????0???00?01?1001011??0???1

Patagopteryx ?0???????????????????????????????????????0010???????????????????0??????????????????????????????2????0??????????????????????????{0 1}????????0?1???????????211101?2????????????????2?010001??10121301001?000??????1??????????1?????????????????????????????????1??????0?01????0????????????????????????2????????2??11??0?????????00????????????????????????????????????????????00??1????????????0??1??????????00?1?????00??11111000???????1000000????????0??????000????011?10???{0 1}?0?0???0000001{0 1}0001?{1 2}00???30????????2000000?0010????0?000{1 2}100110000?{0 1}?100?1?100??0?0?0?001????????0?0010?

Rinchenia ?01?0???0??????????0111?1????1011?1012??00???0000?????00??1??2111001100??111?1????????1??????1??1???1???1???0?????210????????121?1??00000???10?0?01100?1??0??1?????????????1????????0??????0?0???????0???0??01??2?00????????????10111???11?1110?012?10???????????????1???????????????????????????0?????1?????????????????????????0?????1???0?????1110011????110??0???????00????????00???00?????2??010????????10?0?0??0???0?????????0????????????????????????????????????????????????????????????????????????????00???0?00???????????????????????????????????????002?0????????????????

Rahonavis ???????????????????????????????????????????????????????????????????????????????????????????????011111?0?1??1?011?12?12?????????0?0??11??011??????0?01111110011102?002?101110?12111000100001110110110000????0?1?????0?????0?0????????????????????????????0???1111?010???130???????????????????????0211011101?01111011?????11?1111100??01??????1?11?????0?00??????0?001???1??0011???????????????????????????????????????????????????????001000{0 1}0?????????????2??????????0??????????????????????010100?????????????000000?00000001000000000000000?010??0????????01??????1????????0???00?

Sinosauropteryx ?01?0?????????????0001???1??0?0????0000??0???00?00???????????000?0?0?????0?000101010?00??01??100????0?0?0????00?11?00000102????00000000?10??0100?1000020??????00100?100?11?0??00000?000?001000000000?011??000?000000????0?00000000100100010100001?0???0?00001101?111000000000?100001000100000100000000101?110000100000?10001000100?0000001100000?0???000000000000??00?000??00??0000?10?????0?0100?00????????????0????????0101??????00?0???00{0 1}0?0????????????0??0?0?0?????????0?0???0?00????????????1??0000???00000?00000?0??????00??0??0??00????00000????????00????000????????000?0?0

Shenzhousaurus ???0??????????????210001?100?0??1??000??00?000?0???????????0?0000000??0??0?1?12???1????????????0????0???0???00000???0?????????????????????????0010010000??110?00100?000000100?00001?0????????????????00??1???0011????????01020?100?00?100?0000????0?????00??1100???????????0????00?20?000?0010?0201?10?0?????????????100?00?0?0?00?????0???01?0??0?0??0000??00?00?0???010??000?0000????????????00?00???????????????????00???????????0?0?10?0?0?0???????????????????????????????????????????????????????0??????0000?00000?00?000???????????????????0??????????????00?00??????????0???0

Sinornithomimus ?0111????1???????021011?0100?0001?020?0000?0000000??000????0?0?00000000?0001?1????????001?0011?00???0?0?1?10???0?00?00100?2????01020012??0??01?0100100000?110001100?000?00100000001000??00100000203?0000011111?12111???000?0??1100100110010100?01?1?00????111????101111010001?100112?0000000110020101010111?0???10??01100000000200000?0011?0110010??00000?0?10??0?01?0????????????????????????????????????????????????????????????????????????????????????????????????0??????0????????????????????????????????0?????????????????????????????????????????????????????????????????0???0

Struthiomimus ?0111?110??0??101021000101111012110000000000000010000001?01??000000001020001?1????????001?1011000010?0001010000000000010012????01120002010000?10100100000011000110010000000000000011000100100000203?000011111101211100100??0??110010012001010010101?10????111100010001101000111000120000000011002010101011110???000001100001000200000?0011?0110010?00000??0?00100?01?0000000000000001??0???0?11????0??????????0?0?????000??????00??0?000?000{0 1}0?0???????????20??0?0?00?01?0?000000??0000000000??0???0??00000??0000000000000000?0000000000000??0?0200?1002??0??0??0?00?00?????????00000

Segnosaurus ??????????????????????????????????????????????????????????????1??1?0000?000???0100001???????????????????1???0??????????????????0?10?0?00???????0?0?20021100102?0210?0?1020?0????????00??0000?0000?21?0??00???1??0?0????11012111???????????????????10????01???????1000?0??0???????????????????????110100?0?010011???01????10?001??11?01?0???????01??????000?????10??0?1??1??00?0??????????????0???????????????????????????????00?????????????????????????????0??????0??0??????0??????????????????????????????????00??000??10?????00??0?00000000??2??00????????????????0???????????????

Shuvuuia ?011000000000?112011000?0?0??0?111?0000100001010010010???1?1?000?00000021000?021???00??011111101110100??1?201012012000100?1000?00020003021??122011000?1?1011?2000?00021032?0??21010011101211{0 1}0003010000110000110000001001?1??0?100100??00101001011??00??10?1?????010000000?0??1001?000???000110???1???1?211?000?1000011000??0002?001100012101101?0?0?000000?00?01?01?00011100?000000110000?0?1{1 2}00000??10100??1??0010?01?1000??{1 2}0???10?001000?0?0220?0??0???20?10?00001011??001000?00?0000?000?????????300?0??00?00?0?0?0000000100000000?000000?000?00000100000?01010001???????1?????0

Similicaudipteryx ?0110?????????????00010??10?0000??0012210001100?0????????????2?01000?10??100202???00?20??????10010??1?0?0???01??00210?111010??000010001?00??100010010021??????02200?111?1??0??1??0??00???0??00001000?000000????00?0????????02110001?110001?1001?1?1?10???0001110?11001111010??110000??0100000010101010111011000010000001010?00010000000000100101?????0?1000?1101000?1????????????????????????????????????????????????????????????????????????1?????????????????????????????????????????????????????????????????????????????????????????????????????????????????????????????????0?????

Saurornitholestes ????????????????????????01110???????????111????????????11????0???00?????00???010111000?11000120011111011011100?1011?111??????????01111??????1??0?0110221?1?111??????????????????11??0????0??001101??00???00??1??00?000????0000??????013????0??00??00?1?100011????11001??0?1?????0?????????11001??0??1???????????1?21??00?10?010??0?????0??0?0?01?0???0????01??????????1???00000????1101????0?0??????0???????????????????00000???????0????????????????????????????0??001?????????????????????????????????????????0?????????????????????????????????0??????????0???00111????????0??????

Sinornithoides ?0??0?????????????1?000??1???0????020?2???????1??????????????00??010???????000110101??????11?1001???????????0011?2?102101?2??1?0?011101?00??1000000???21??01??0?200??11?1?100?11110?????0??10000111001?0??00?1??0000???00???301100100110?1?100??1??00?1?101????0?11?0111101001110001000000000000???01?1111111000012110000101??0210000000001?010110???00?000?000?0?1??100?0?000?000?????????0?010000????????????????????00??????00??0????????00?0?????????0?20??0?0000??0???0?000???0?0?????????0??010?0000???0000??00??0?00????000000000000000?020000????????11????1?010100??0??{0 1}???0

Saurornithoides ?01???1??1101???0?110001?10000???????1?????????????????1?010?1000010??1????0001101010????????1???0??0?????100??1????0?????????????????????????????????????????0020?0?0101?10??11110?????????00??211?01????????1?0?????0??00?30?000100110010000??1?0???1?10??1??0?????????0??????0?????????????????101??1??1??01001210????10???0???0????0??00?????000??0???010000?111?10?1????0?00?0?11?????????0??000????0?1000?0?0???????????000?????0010?0????????????????????????????????????????????????????????????????????00???????0?????0000000000000?0?02000000010100110?1110?1??????????????

Sinusonasus ?0????????????????01010??11100?????20??????????????????????????0?01????????0?01110?1?????????????????????????01??2200?????????????????????????????????2???????002?????1??????????????0????1?00??2110?1??????????0?????????0?30110010011001?100????0?0?1??0?????0???????????????????????????????????01?111?11?00001210????1???1021?0??000???0?1???0????00?????0???1?1?1????????????1??????????????????????????????????????????????????????????????????????????????????????????????????????????????????????????????????????????????????????????????????????????????????????????????????

Sinovenator ?0???0110010011110010101?11100?01102?12000011?1?1?101001???0?000001??????00000111?01?????11?11100011010000100011022??1?????????010111???????100??00?0?211?0101?02000211021100?11110000??1011000111??110?000001??000011???0??30?100?00?000??10?1?11?0??10?001111??1101???00??????0???0???0000000???201011101?1???1011??0??1010112?00??000??100??110???0??000100100110??0?100001000?0??1?00?00??1000000000?000000?000??000001010{1 2}00??00?001000???????????????20000??00010?????????????????????????????????????????00?100000100000000000000?00?00?02000000010001?101011000???????0??????

Sinornithosaurus 0011?????0?????????0000??111100???10021110021?1??1??0????????0000000?11??0?0101010{0 1}00?0??????1??1???????0???0011??1????1??01?1001011110?00??1?00000?0?2?1?01?1002?0?211021?2????11???0???11000111100?0?0001001??0000???0?00?{0 2}00100100130010100101?000011000?????00101111211001111000010011{0 1}100{0 1}01?21111?1?1000012021{0 1}0000121?1121000001000100101?0??0??0001100100100110010?01100001110??0????0100?00?????0??000???0????00000??000??00?0????0?0??0????????0?{1 2}0000???00010?0??00?0??00???????????????????0000??00?0000??0?000????000??0?000?00????10000????????0??000111?1100?10001???0

Songlingornis ????????????????????0?0??????????????????????????????????????00????????????000?0???0??????????????????????????????????????11??{0 1}???3?????????????????????????????????????????????????????????????0??????????0????00??????????????????????????????????????????????1???2????0??????????????????????????????????????????????????????????????????????????????????????????????????????????????????????0?01????????????????????????????????????????????21??{1 2}?0000?010???1?101???????????????????????????????????????????????????????????????????????????????????????????????????????????????

Tanycolagreus ??00????????????????0?0??????0????0010???????????0??????????????????????00000?????1??0???????????010000????????0??0????????????000000000100???00010???????????????1??????1000000001000000010000000?00000??0???1??000???00????????0?1???00???0?????????1?????110??10001111000010100010001001000001????01010110000100000011?01?0?1??00000?00??0?000?????????0???????00?1???????????????????????????????????????????????????????????????????????????????????????????????????????????????????????????????????????????????????????????0???00?????????????????????????0???????????????0?000

Tarbosaurus ?1000??0??????????0?00110101000000011012???2110?001100010?0000000000?11?100000001010110???00?000????100?0???000?0000000??0?????000000010000???0110010?10??100000101?011?00?0000?00??000?00100000200000001000000000?0?0?0010?0000000101011000000100010?1100000????10100?00000??0000?1??0??000000??0011010101100000?000???0000000000?00?00?00{0 2}01??000?00000000{0 1}0000??1?000000?00?000?110111111?1000000000000????0?000???000??????????00?00?00????0???????????2???0??????01?0?000?0???0???00?????????????01??????0?00??0?00?0010????0????00???0????0?0?02000??000?000100000??????0?000?0

Tyrannosaurus ?1000?0110000?002100001101010000000110120102110000010001?00000000000010010000000101011010000000000101000000100000000000??0????0000000010100???011001031000100000101101101000000100100001001000002000000010000000000000100100000000010101100000000001011000000000010100000000?10100?1000??00?0????000100?1011000010000??1000100000000000010020100000000000?0000000?01?000000000100001101111111100000000000000000?000??000001011000??000001000{0 1}0?0????????00020010?0000101?0?000000?00000000000010??0?????000??00?00?000000001000000000000000000?000??02000??000?000100000110?000?00000

Therizinosaurus ???????????????????????????????????????????????????????????????????????????????????????????????????????????????????????????????001000?0?00??0?000?0???????????????????????????????????????????????????????0??????0?0???11????????????????????????????????????????10?0?00??0?010??000101????1?10????????????????????????????????????11???0???0?10?????????????????????????????????????????????????????????????????????????????????????????????????????????????????????????????0????????????????????????????????0?????????????????????????????????????????????????????????????????000?0

Tianyuraptor ??11????????????????0???0111?????????2???0?????????????????????????????????0?0{0 1}01010??0???0?????????0?0?0?1??01?0111??????01??00?011110?001?1100001102?1??0?11022?0?211021?0??1?110?????????00?11????000??00?1??00?0???000??000???????300?0?00?0??????01??0?111?0110011110100?1101?10?01111100102020101110100???????1000012?0?0110000???000?0111????????0?01?????1?0????1??111?0???????????????00?0?????????????????????????????????0????????0?0??00????00??0?00?0000?0?????????????????????????????????????????00?100???10????????????0?0???????1?????????????????????0000?10??????0

Tsaagan ?01101001000012011200001010100001?100221111200010110000100??00000000?1111000101010100001100??20??????????????0??0???0?011?0????01?1110????????00??????????????????0??????2?00??????????????100???1?????000?00??00?00000???0?000000100130000000001?0???0?00?1???0?1???1??1??0??110??1??001???????0????01?1?1?????????????0???????0?0????00??0?????0??0{0 1}00??010001?1????10?0?????0000110?0000?0??000000????000000?000??000000000000??00??????????????????????20000???00?????????????????????????????????????????????????????????????????????????????????0111000??000011?0??????????????

Troodon ???1?112?1101000001???0?01?100?????20120000210?0??01100????0?10??01??????????011010100???1111100101101111?1000?1020??11?????????????1??10?????00?010???1??????002??0?11010?0?01111000???00100000210?01????00??1?0??0100???0?30??????0?1???????10?0?0???110??111????????1?01?0????????????????????????????11?101001210????????101??0??000??0?????10???0??0001?????111?1001?0???0000?0????00???????????????001??0?00?????0??1?1??????00??????????????????????????????????????????????????????????????????????????????????????????????????????????????00000101001?0?1110?1??????????????

Utahraptor ???????????????????00?0???????????0002?????????????????????????????????????01???101??1????00110011110?0??????0?1011?????????????101?1?????????????????2???0?01001??0????10????1111000??0?01000100???10???????11??0???????0???????0?0???????????????????10??1000?????0??????????????????????????????01?00?????????????????10??00???????????0??????????0???????????????1????0???0????????????????0???0??????????????????????????????????0000?????????????????20000?00000??????????????????????????????????????????00?????????0000000000000000????0?0???????????????????????????????????

Unenlagia ?????????????????????????????????????????????????????????????????????????????????????????????????111111111110??1????1??????????0?0??1001???????????11111110011102000211?21120?1111000???001000111100000??????11????0????00?0????????????????????????????????101??010?11???????????????????1?0????0?11011??1?0???1?11?????122110210?000?????????11???????00??????0????1??1??1010??????????????0????????????????????????????????????????0000?0???????????????1??????????10?0?001000?0000????????????0?????????????00000?000000000000000000000000?0?????????????0???????????????????0???

Velociraptor ?011010010000120112000010111000011100221111200010100001100100000000011111000101010100001100012001111101111100011011112111001011011111001001110000010022111011100200011112110011110000000001110110100100000000110000000000000000001100130000000001100??0100?1?010011001111010?11100?1000000110000202010110011000?212100001121010110000010000001011000??00??0000010100111010000010000110100000001000000000?000000?000??00000000?000??00000000000?00??000?000020000?0000010?0?00000???000000?000010100???00000??10000?000000000000000000000000000?01000010{1 2}1100001000011100000?100?0???0

Vorona ?????????????????????????????????????????????????????????????????????????????????????????????????????????????????????????????????????????????????????????????????????????????12?01001110101112110???00???????????????????????????????????????????????????????????????????????????????????????????????????????????????????????0????????????????????????????????????????????0???2????????????????????????????????????????????????????????????????????????????????????????????????????????????????????????????????????????????????101011110001?00000?????????????????????????????0??????

Xixiasaurus ???????????????????100010111?0?????2?12????????????????????0?10??01????????00020??01?0?????????????????????????????????????????????????????????00?1????????????????????????????????????????????????????1???0??????????????0?0???00?0?1?00???0??????0??1?10???????????????0?????????????????????????????????????????????????????????????????????????????????1?0????????00???????0000??1?0???????00000???00??????????????????????0???????????????????????????????????????????????????????????????????????0?????0????????????????????????????????????0?????????????????????????????0????

Xiaotingia ?01????????????????1?1???1111000???0?2??????????11????????????00?01??1???00??020??0???0??0???1?0????0?0?01?????1??????10?12???01?01111??0???1000101011?1?10?11??2?????102?1???????????10???????1?11000101000?1??0?10??????102?110110?1??011100?01?0?00???0?1111?1011?111211001110001??001011001020201?????1??00110011100012?0?1??000?00000??0??1????00?000110010011?1?0????00??00?1???????00?010??00????????????????????0000???????0?00?1000???0????????0002??????????00?0?000?0???00?0????0???????????01000??0000???0?000?????????????????0?0?02?000????????0???????1?1100?10?10??00

Yixianosaurus 0?????????????????????????????????????????????????????????????????????????????????????????????????????????????????????????2??????010010??0??1000{0 1}00????????????????????????????????????????????????????0??00?????0?????????????????????????????????????????????????10?01?01??11100?10?0??0?10010?????????????????????0000??????????1?????0??0???????????????????????1?????????????????????????1????????????????????????????????????????????????0??????????0100???0?????0?????0?0???0???0???????????1??00????0?0???????????????????????????????????0?????????????????????000?00??00?00

Yanornis ?01???????????????11?10??????0?11??0??2??0?????010???????????00000???00????00020??00000???11???00?????0?2?1???????0???111?110?001031110?011?1?0000?????????????00???121?32?01?2?0100?1???0121{2 3}0000100000??011???00?0???01?1?200001?0???0011100??1?00?00?001111??10?022113011??1110?0011??1111101?????01?1?1201?010000???0??????1??00000000100?0??0??10??001?11???????1??????0?201?1??????????0210001??????????10??????1???????????????001?00???0210?{1 2}??100001010?1?1??111??011?0101?0000?0?????021{0 1}101311{0 1}110{0 1}10?0?1?0??000?????0??00?110{0 1}?1?0??1?100????????0?????????1100?11?00???1

Yixianornis 101????2?021??1???2?0?01?????0?1?1?????????????????0100??????000100???0?00?001?0??10?01???1???0000??1?0?20??01??0022???11?1100001031110?00101300{0 1}0100021?101?2?020?022103222?0210?0??1?????2130100100000??00?0??1000?2??1?1???0???1????001?????0???????????1????101022113011??1110?0?111?1??00?1?020101?1?1201111?0?0????102?012??000?000010???1?0???1???01?????????1???10??000????????????0?0210001??????01??1???????11{0 1}??????0???102001000?11021??{1 2}??100001010?11101111100?110???0?000???0?01021{0 1}10?3120110{0 1}10?00100?0000?101??????1110{0 1}?1?0??1?100??????0001??01????0100?10??0?1?1

Zanabazar ?0110?12?110100?0011000??100?000???2012000?21??0??11100??????100001???1????00011010100??????????????????1?1000?1020?1????????????????????????????????????????????????????????????????????011????2???????????0??00???110???0?30?1001001100100001010000?1110?????0??????????????????????????????????????????1??????????????1?????????????0??00?1???0?0????????0000?111??00?????0?00000100??1?????00?00?????001??0?00????????????000??00??????0{0 1}0?????????????????????????????????????????????????????????????????????????????????00010000?000??????0?0000010100111?1110?1??????????????

Jixiangornis 101?????????????????0?0??????????????????????????????????????000?0?????????1??????????0????????00????1??2????02??1?11??11?1?01101031110001??120000011??10?0??2??0?????1??11?1????1?00???101?1101001000?00?00????1000????0?????0????0???00?????????10???????1????10?012?130????1110?0??1??100000???????1?1?1?0111200?10010100???1??00?01000?001???0?????0??1????0????????????0????????????????02?0?0????????????????????????????00????00?0????0?0????1??0000000???1?10??0?0?001000?00?000?00????????10?10100?000?0?????0?000?00????000??0?????0??00100????????0?????????1100?110??1??0

Epidexipteryx ?0???????????????????1???????0?1???012???0?00???00????????????00???0?1??00002022??10?20???0??0??0???0?0?11??102???22??100?00???0?01?011?0??????0200011?10?????020?0?011?02?0?????????0???01?00??0?100000???0??1?1?0????0000021000010?0?0?11?00101?1???0??00?111??111011120?????????????????0?00??01??01?1?1?0????????????10?0?0100?000?0??1?1????0???0??111?11011000???????00?0????????0??00?01????0??????????0?????????????????????000??00?00?0???????0????0?????????00?????0?0???0??0?????????????????????????00??00?????????????????0???0?????0??0????????????01??0?????????00?00?

Jinguofortis ?0?????????????????1????????????????????????1???1????????????000???????????0??????00????????????????0?0?21???1?????2???11?10?10??131?10?00??1300000011?1??????100?0?2?1?21221??????????????211??0?000000??00????10?????01?????0100?0???0????00????1??????10?111?1010?2113111??1110?0??01111100?1?00??01?1?1?011120001??1000?0?01??00?00000??0?0??0???????01?????1???1???????0?????????0??????02???????????????????????????????0??????20??0??1100??????00000???0????1??10?????1?0???0?00??0?00?????????21????001?000???????0?????????0??0???1?1??2?100????????0?????????1100?11000????

Zhenyuanlong ?0?????????????????00?0??1111?????1012?1?0??0????????????????00??000?11??0?010?0??1??????????1????????0?1????01???1?1??01?00?????0???1??00??110000??02?1??01??0220??111?2112?????????0????1??0??011000000000?1??0?0????000??00?000?001000?0?00?0??0???0??00?11?0??10?11111100?1101000?0111110010202?101?1?1?00011121000001??01011000?01000??0???10?????0000100?0010?1?00?0?00??00001?0?0??00?010??00????????????????????????????????0?0??00000?0??????00??????????????0??????0?0???0?000???000????????01000??00?00?0???0?00????????????0?000??0?10000????????01?0?0??1????????001????

Jianianhualong ?0???????????????????????1110??????2?1????011????????????????0000010011?00???0100?01??????1??1??????0?0??????011?{1 2}20?2000?2???00?01??11?01??1100000???????????0020??211?????0???????????????00000?10?111??00????0???????????201???1?010001?1??101?0000???00?11????11?11110100?1101010?01000000002??11???1?11?0001?21?0000100?0111??0?00000?001???00?0{0 1}?0??0100?0?1??1?0?????01?0001??10???0?001?0???0?????????0?????????0?????000???000??0???0?0????????0?02??00?0????00?0???0?0??????????????????????000?0???0?000??????00????????????0???0?0??20000????????????0?????11???110?1????

Anchiornis 00110?1?0?{0 1}0?????011010??1111001??00?2?10001101?1101?????????0000?10?10?00000020??00100?101??100?0??010?011??011012101000?2???011011110001??10001011021101000102200?211011120?1111?000???0100001001000000000?1??0000?11000102001001001000101001011000?0??011111100101110201101110001010101110010202100111?11000110010000010000021000?0000010010110000000001100100100110110?00100001?11000?00?0100?000?????????0?????0????0????000??00000?00000?0????????00020000?0?0??00?0???0?00?00?000?0?????????10?01000?000?000000?0000?0??00?000?100000?00?00000????????0??1001?1?1100?100110000

Jeholornis 10??0?????????????01010?0100?0?0??0012?1?0000???00????????????00000???020001?{0 1}22???0??0????????00???0?0?11???02??1211?101?10?1111031110001??120000001121??0???100?0?111021101???11?00???10101111001000000000?1??1000???010102?0000100120010100101?1000???01111111010121130110?111000011011000000002??01?1?11011120001001010000111000000000100101?0??0?00001???0011?0110?????0??0001???0????0?02?0?00???????????????????????????00??00000000000?00???1?0000000000?1?1000000?001000?00?0000000001000010110100?0000000000?00000001000000010000100?000100????????01?10???1?1100?1100011?1

Sapeornis 10110?????21??????01010??0?000001?00022100000?1?000??????????000000?110?00000022??00000????1???2000?110?211?002?00021?100?2???201031110101111300001011210?00?2100?00111021101???010000???0121111000000000000?1??1000???010102000001000?0011100101?10?00?001?111?00101211311101111100011111110011?02??0111?11011120001??101000001?000000000100?0110??0?0?001?1100100011?1100?0?00001?1100?100?0200?000?????????????????????????100??0000000000100????????00020000?0000?0000?001010?000000?1???010?1{0 1}10?01100??00?000000000000001000000?10000100000?000????????01?1011?1?11000110101111;

END;

BEGIN ASSUMPTIONS;

TYPESET * UNTITLED = unord: 1- 14 18- 31 33- 38 40- 43 45- 60 62- 71 73- 104 106- 107 109- 115 117- 119 121- 126 128- 136 138- 150 152- 157 159- 160 162- 164 167- 168 170- 187 189 191- 219 221- 261 263- 264 266- 270 272- 275 277- 284 287- 288 290 292- 296 298- 299 301- 304 306 308- 319 321- 329 331- 366 368- 382 385- 386 388- 396 398- 403 405- 421 423- 427 429- 430 432- 437 439 441- 461 463- 466 468- 480 482- 486 488- 493 495- 496 498- 500 502- 510 512 514 516- 517 519- 521 526- 528 530- 533 535 537- 565, ord: 15- 17 32 39 44 61 72 105-108\3 116 120 127 137 151 158-161\3 165- 166 169 188 190 220 262-265\3 271 276 285- 286 289 291 297-300\3 305 307 320 330 367 383- 384 387 397 404 422 428-431\3 438 440 462 467 481 487 494-497\3 501 511 513 515-518\3 522- 525 529 534 536;

END;
